# Canyon Wall and Floor Debris Deposits in Aeolis Mons, Mars

**DOI:** 10.1029/2021JE006848

**Published:** 2022-01-27

**Authors:** M. N. Hughes, R. E. Arvidson, W. E. Dietrich, M. P. Lamb, J. G. Catalano, J. P. Grotzinger, A. B. Bryk

**Affiliations:** ^1^ Department of Earth and Planetary Sciences Washington University in St. Louis St. Louis MO USA; ^2^ Department of Earth and Planetary Science University of California, Berkeley Berkeley CA USA; ^3^ Division of Geological and Planetary Sciences California Institute of Technology Pasadena CA USA

**Keywords:** Mars, geomorphology, debris flow

## Abstract

Aeolis Mons (informally, Mount Sharp) exhibits a number of canyons, including Gediz and Sakarya Valles. Poorly sorted debris deposits are evident on both canyon floors and connect with debris extending down the walls for canyon segments that cut through sulphate‐bearing strata. On the floor of Gediz Vallis, debris overfills a central channel and merges with a massive debris ridge located at the canyon terminus. One wall‐based debris ridge is evident. In comparison, the floor of Sakarya Vallis exhibits a complex array of debris deposits. Debris deposits on wall segments within Sakarya Vallis are mainly contained within chutes that extend downhill from scarps. Lateral debris ridges are also evident on chute margins. We interpret the debris deposits in the two canyons to be a consequence of one or more late‐stage hydrogeomorphic events that increased the probability of landslides, assembled and channelized debris on the canyon floors, and moved materials down‐canyon. The highly soluble nature of the sulphate‐bearing rocks likely contributed to enhanced debris generation by concurrent aqueous weathering to produce blocky regolith for transport downslope by fluvial activity and landslides, including some landslides that became debris flows. Subsequent wind erosion in Gediz Vallis removed most of the debris deposits within that canyon and partially eroded the deposits within Sakarya Vallis. The enhanced wind erosion within Gediz Vallis was a consequence of the canyon's alignment with prevailing slope winds.

## Key Points

- Debris deposits on the walls and floors of canyons cut into Mount Sharp were a consequence of late stage, water‐related events
- Wind‐driven erosion degraded many of these features, including differential stripping of wall and floor deposits to generate debris ridges

## 1 Introduction

**Figure 1:**
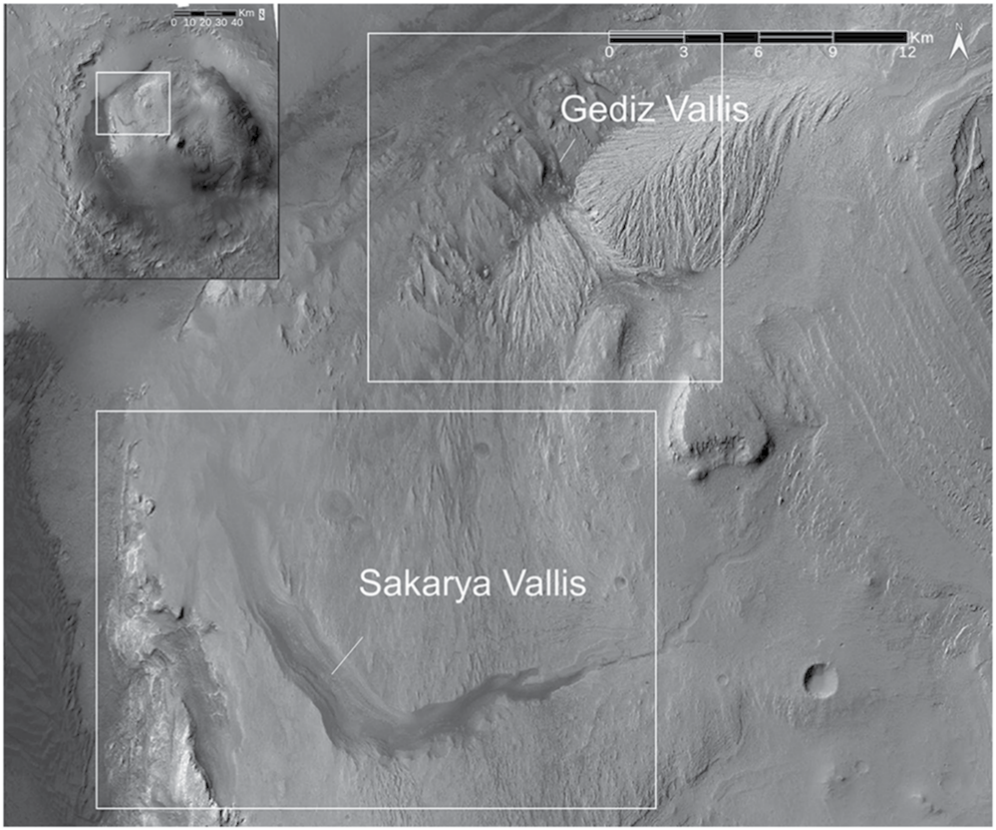
CTX‐based mosaic views that cover Mount Sharp and the canyons that are the focus of this paper, Sakarya and Gediz Valles. Regional view is in the upper left covering Gale Crater and Mount Sharp, with the white box delineating the overall study area. White boxes in the detailed view delineate areas shown in detail in Figures 2 and 7.

Aeolis Mons, an 85 km wide, 5 km high mound, informally named Mount Sharp, is located within the 154 km diameter Gale Crater (Anderson & Bell, 2010; Figure 1). Partial erosion of lower Mount Sharp has exposed sedimentary strata, with fluvial and deltaic sandstones, and lacustrine mudstones (e.g., Grotzinger et al., 2015). Sulphate‐bearing strata overlie those rocks, with relatively bright strata capping the stratigraphic section (e.g., Anderson & Bell, 2010; Milliken et al., 2010; Sheppard et al., 2020). Based on remote sensing image data, exposure of the overall stratigraphic section that underlies Mount Sharp is due to an erosive combination of mass wasting, wind, surface runoff, groundwater, and possibly ice‐related processes (e.g., Le Deit et al., 2013). In contrast, Curiosity images do not show evidence of glacial landforms or deposits, suggesting ice‐related activity has not been an important component in shaping Mount Sharp. Water‐related activity that post‐dates formation of Mount Sharp includes the presence of three lakes within the surrounding plains (Palucis et al., 2016), with evidence presented in the form of fluvial channels, deltaic complexes, and alluvial fans (Grant, et al., 2014; Palucis et al., 2014, 2016). Large debris flow deposits have also been proposed on northern Mount Sharp (Anderson & Bell, 2010; Le Deit et al., 2013).

Erosive landforms dominate the morphology of Mount Sharp. These include Gediz Vallis, on the northern side, and Sakarya Vallis on the western side, as well as other unnamed canyons (Figure 1). Both Gediz and Sakarya Vallis extend from the upper portion of Mount Sharp, through sulphate‐bearing strata (e.g., Sheppard et al., 2020), to the floor of Gale Crater. Both canyons exhibit debris deposits that appear to over fill (i.e., they are ridges) sinuous channels cut into the canyon floors (Bryk et al., 2020; Hughes et al., 2020). Poorly sorted debris deposits on the canyon walls range from those contained in chutes cut into wall rocks, to ridges standing above the surrounding wall strata. Where not obscured by modern wind‐blown sand sheets, these materials merge with channel‐filling debris deposits on the canyon floors.

The intent of this paper is to understand the formation mechanisms and modification of the debris deposits within Gediz and Sakarya Valles. We proceed by first describing data sets and methods. We characterize the overall morphology of the two canyons, and then focus on debris deposits and associated features (e.g., chutes, lateral ridges, etc.). We consider the sulphate‐bearing rocks cut by the canyons, with implications derived from the highly soluble nature, and likely low strength, for the generation of blocky regolith conditioned to be eroded and transported downslope. We end with consideration of the role of fluvial processes, landslides that become debris flows, and dry landslides in generating the debris deposits and implications for hydrogeomorphic event(s) that post‐date formation of the canyons.

## 2 Data Sets and Methods

We used a multi‐dataset approach to pursue the analyses outlined in the Introduction (Table 1). We pursued mapping and morphologic characterizations of relevant deposits and associated features using the best available image data. We utilized Mars Reconnaissance Orbiter (MRO) Context Imager (CTX, Malin et al., 2007) mosaicked data and associated digital elevation models (DEMs) for Sakarya Vallis. The individual scenes for this mosaic are listed in Table 1 and available through the Planetary Data System Cartography and Imaging Sciences Node (https://pds-imaging.jpl.nasa.gov/). MRO High Resolution Imaging Science Experiment (HiRISE, McEwen et al., 2007) images and HiRISE‐derived DEMs were used where available. The HiRISE DEMs covering Sakarya Vallis were generated using the method described in Mayer and Kite (1903), and are publicly available (Hughes, 2021). For Gediz Vallis, a HiRISE‐based mosaic and digital elevation models (DEM) were used for the analyses, and are publicly available on the USGS Annex (Calef & Parker, 2016). A Mars Express High Resolution Stereo Camera (HRSC) image mosaic and associated DEM over Gale Crater were also utilized (Gwinner et al., 2009, 2010; Jaumann et al., 2007). The individual scenes are available through the Planetary Data System Geosciences Node (https://pds-geosciences.wustl.edu/).

**Table 1:** Datasets Used in Analyses

Instrument	PDS (Planetary data system) product ID	DOI
Context Camera (CTX)	G04_019843_1746_XI_05S223W	10.17189/1520266
	G05_020265_1746_XI_05S223W	
	P04_002675_1746_XI_05S222W	
	B21_017786_1746_XN_05S222W	
	D02_027834_1748_XN_05S222W	
	G04_019698_1747_XI_05S222W	
	P01_001422_1747_XN_05S222W	
	P18_008002_1748_XN_05S222W	
	P04_002464_1746_XI_05S221W	
	B18_016731_1747_XN_05S221W	
	D07_029891_1747_XN_05S221W	
	D13_032159_1745_XN_05S221W	
	P03_002253_1746_XN_05S221W	
	G10_022190_1746_XI_05S221W	
	G03_019566_1746_XN_05S220W	
	G06_020489_1746_XI_05S220W	
High Resolution Science Experiment (HiRISE)	[ESP_024234_1755, ESP_024300_1755	10.17189/1520179
	PSP_009650_1755, PSP_009716_1755,	10.17189/1520303
	PSP_010573_1755, PSP_010639_1755,	10.17189/1520227
	PSP_009505_1755, PSP_009571_1755,	10.5281/zenodo.5114387
	ESP_011417_1755, ESP_011562_1755,	
	ESP_018854_1755, ESP_018920_1755,	
	ESP_023957_1755, ESP_024023_1755,	
	ESP_024102_1755, ESP_025368_1755,	
	PSP_009149_1755, PSP_009294_1755,	
	ESP_012551_1755, ESP_012841_1755,	
	PSP_001488_1755, PSP_001752_1755,	
	ESP_019698_1755, ESP_019988_1755] (Calef III and Parker, 2016 Gediz Vallis mosaic)	
	ESP_012340_1750, ESP_012195_1750,	
	PSP_006855_1750, PSP_007501_1750, upper and lower Sakarya Vallis, respectively	
High Resolution Stereo Camera (HRSC)	H4235_0000_DA4	10.5270/esa-pm8ptbq
		10.5270/esa-1uqwzjv

We calculated the solubility of reactive components for the sulphate‐bearing strata previously identified by Milliken et al., (2010), Sheppard et al., (2020), and Thomson et al., (2011) to assess the likelihood of water‐induced weathering of wall rocks to produce a blocky regolith conditioned to fail as landslides. Calculations were performed in The Geochemist's Workbench version 12.0.5 (Bethke, 2007) using a previously described Pitzer‐style database (Liu & Catalano, 2016). Volume changes associated with sulphate mineral phase changes, along with likely rock strengths, were used in evaluating the likelihood of blocky regolith production.

An infinite slope stability model (e.g., Selby, 1993) was employed to evaluate the likelihood of landslide failure by incoming pore water for a range of plausible material properties and slopes.

## 3 Morphologic Characteristics of Gediz Vallis

**Figure 2:**
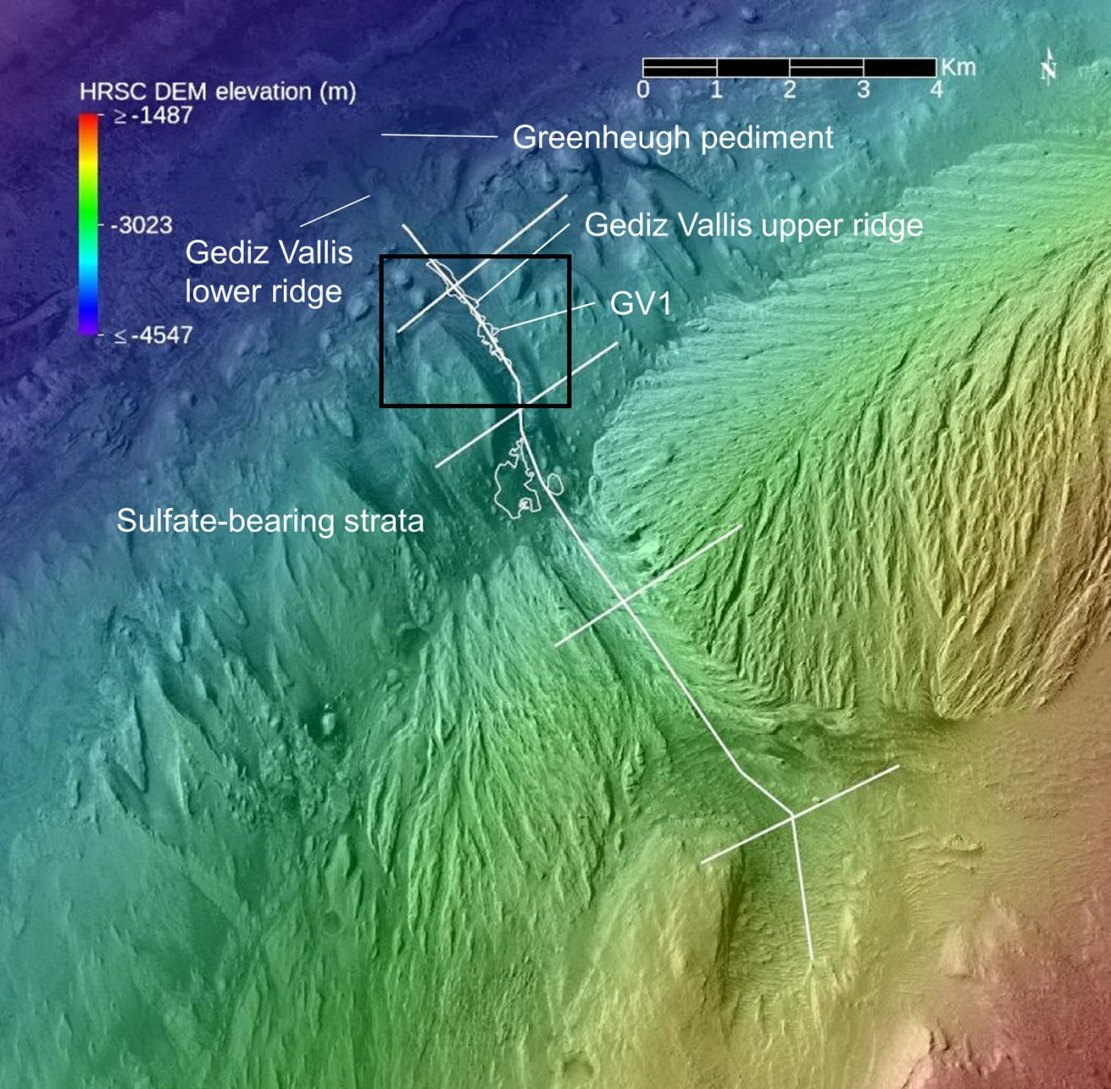
CTX‐based image mosaic covering the Gediz Vallis area overlain with color‐coded High Resolution Stereo Camera‐based elevation data (H4235_0000_DA4). Gediz Vallis cuts down through a light toned yardang unit (Anderson & Bell, 2010) and then a lower section of Mount Sharp dominated by sulphate‐bearing strata. The Greenheugh pediment extends from the lower edge of the sulphate‐bearing strata. The Gediz Vallis upper ridge merges down‐canyon with the more massive Gediz Vallis lower ridge (Palucis et al., 2016). Both ridges consist of blocky, poorly sorted debris. White lines delineate the locations for the topographic profiles shown in Figure 3. Debris deposits within the canyon wall (GV1) and the floor of Gediz Vallis are outlined in white. Black box delineates area covered in Figure 4.

**Figure 3:**
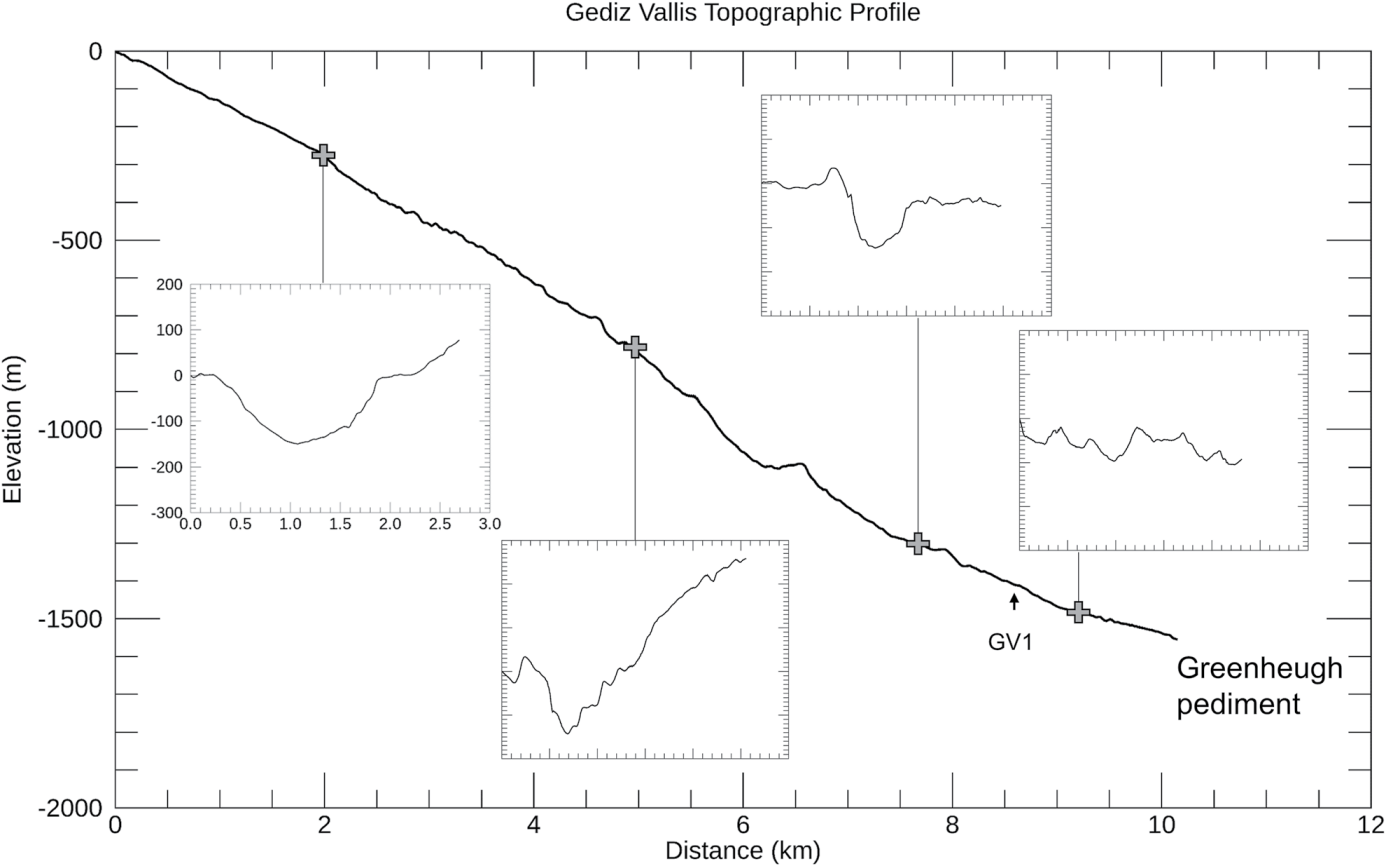
An along‐canyon topographic profile is shown for Gediz Vallis, with cross canyon profiles delineated for key regions. The canyon floor profile has a 5.5 vertical exaggeration. Elevations for the cross‐canyon profiles each have the same width and elevation ranges, with a 4.5 vertical exaggeration. Cross‐canyon profiles run from southwest to northeast. Also shown is the location of the single debris ridge on the canyon wall (GV1). Based on the HiRISE mosaic and digital elevation models (Table 1).

Gediz Vallis is located on the northern side of Mt. Sharp and is ∼9 km long, ∼900 m wide at it widest point, up to ∼140 m deep, with a mean slope of 10° (Figures 2 and 3). Gediz Vallis begins as a broad canyon that narrows downslope (Figures 1 and 2). It extends through the sulphate‐bearing bedrock, and ends upslope of the Greenheugh pediment (Anderson & Bell, 2010; Le Deit et al., 2013; Milliken et al., 2010; Palucis et al., 2016; Sheppard et al., 2020; Thomson et al., 2011).

**Figure 4:**
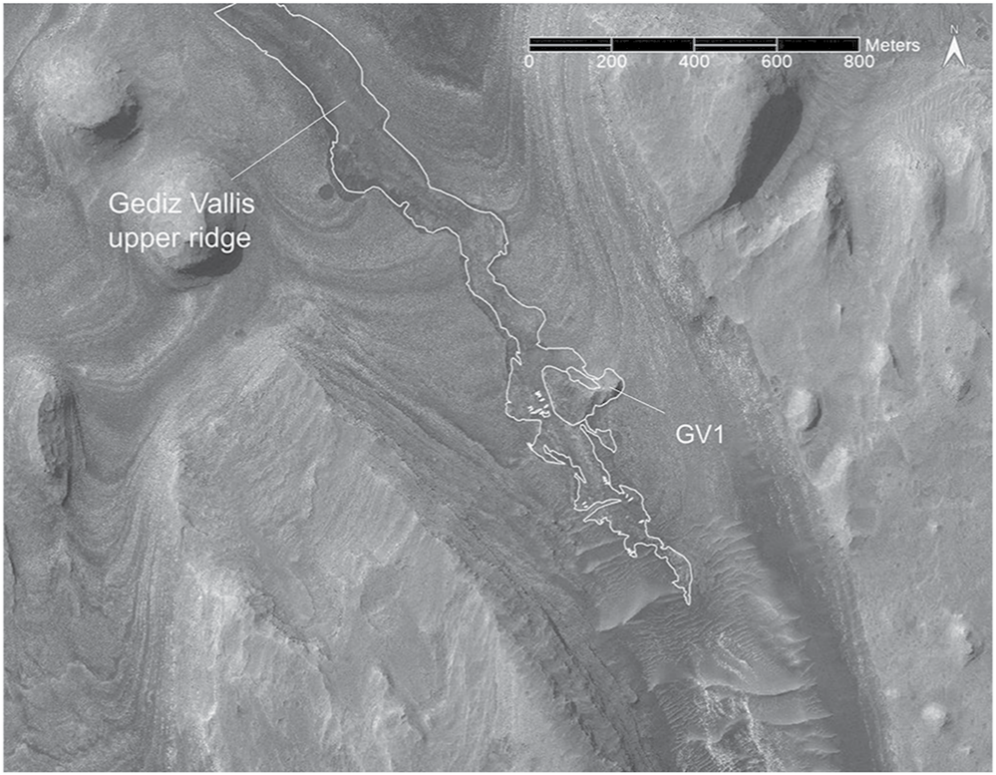
HiRISE‐based view the sulphate‐bearing portion of Gediz Vallis covering the Gediz Vallis upper ridge of debris that over‐fills an undulating channel. Also shown is the location of debris ridge (GV1) located on the eastern canyon wall. The white lines delineate the extent of these debris ridges.

**Figure 5:**
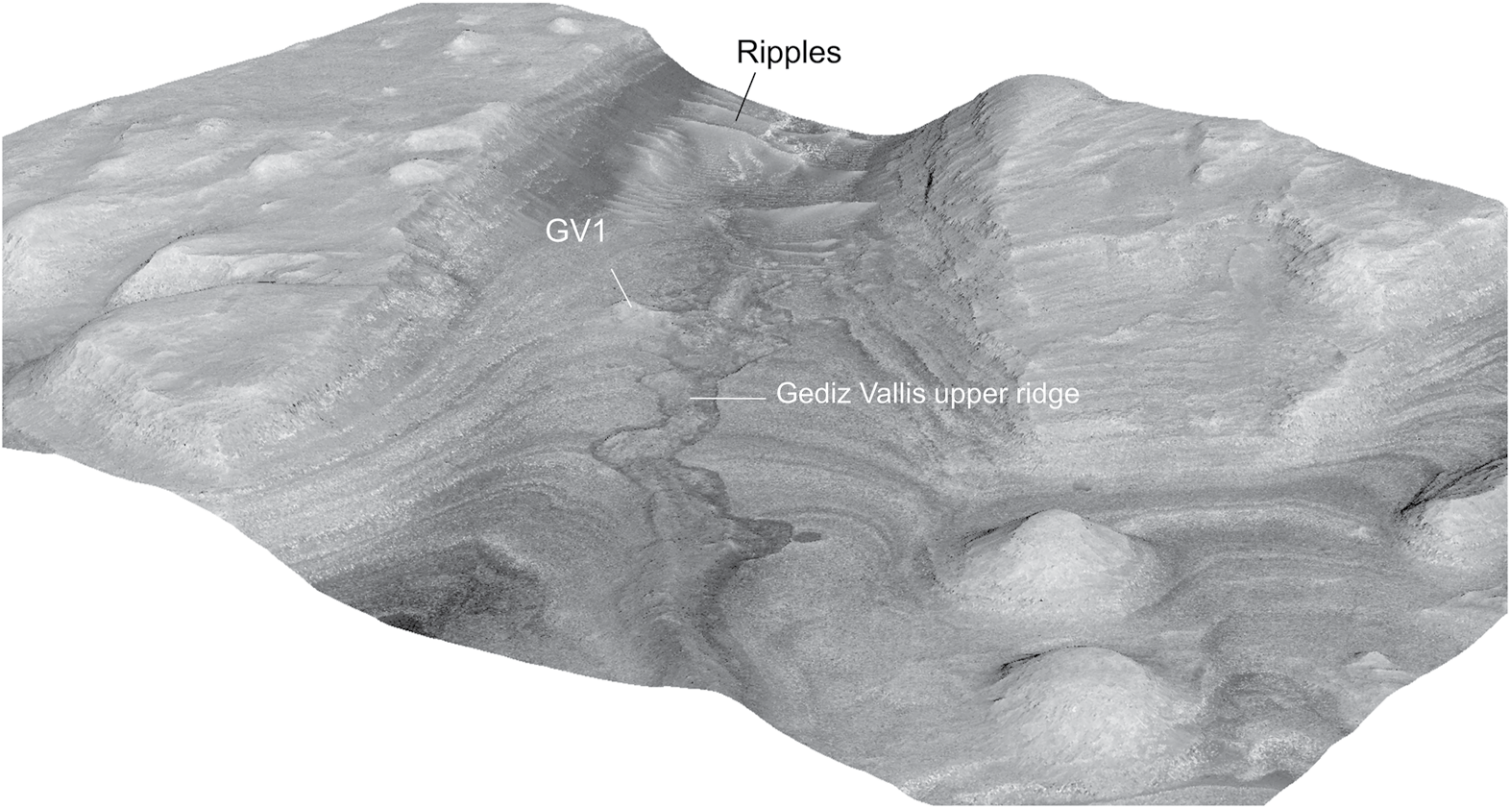
Perspective view of the section of Gediz Vallis located within the sulphate‐bearing section and shown in plan‐view in Figure 4, illustrating the overall morphology of the canyon and debris ridges. Wind‐blown basaltic sand ripples and barchan dunes are evident up‐canyon, with an orientation that indicates mainly uphill transport for sand‐driving winds. The Gediz Vallis upper ridge is ∼100 m wide, and rises up to ∼10 m above the surrounding canyon floor and the present day border of the undulating channel. A 2.0 vertical exaggeration is used for this and other perspective views.

Wind‐induced erosion has shaped the relatively bright bedrock of upper Mount Sharp into linear ridges and valleys analogous to terrestrial yardangs (e.g., Anderson & Bell, 2010; Le Deit et al., 2013). Wind erosion has also occurred on the walls and floor of Gediz Vallis. Wind‐blown sands shaped into sheets and ripple fields cover the upper half of the Gediz Vallis floor where it cuts through the sulphate‐bearing strata (Figures 4 and 5). The lower half of the canyon within these strata exhibits walls with well‐defined layered deposits traceable in some locations across the canyon floor. A sinuous ∼150 m wide channel is located on the lower canyon floor and is over‐filled with blocky, poorly sorted debris (upper Gediz Vallis ridge, Bryk et al., 2019; Hughes et al., 2020; Palucis et al., 2016). The ridge contains boulders up to a few meters in diameter and extends down canyon, ranging from several up to ∼20 m in height above the surrounding canyon floor, and has been interpreted as an inverted channel (Bryk et al., 2019). It transitions to the large lower Gediz Vallis ridge located on the Greenheugh pediment (Bryk et al., 2019). The lower debris ridge exhibits blocky, poorly sorted debris and extends up to ∼70 m above the pediment surface and has been interpreted as a remnant of fan deposition (e.g., Anderson & Bell, 2010; Bryk et al., 2019; Palucis et al., 2016). The pediment is capped by the Stimson formation wind‐blown sandstones draped along the footslope of Mount Sharp (Banham et al., 2021).

**Figure 6:**
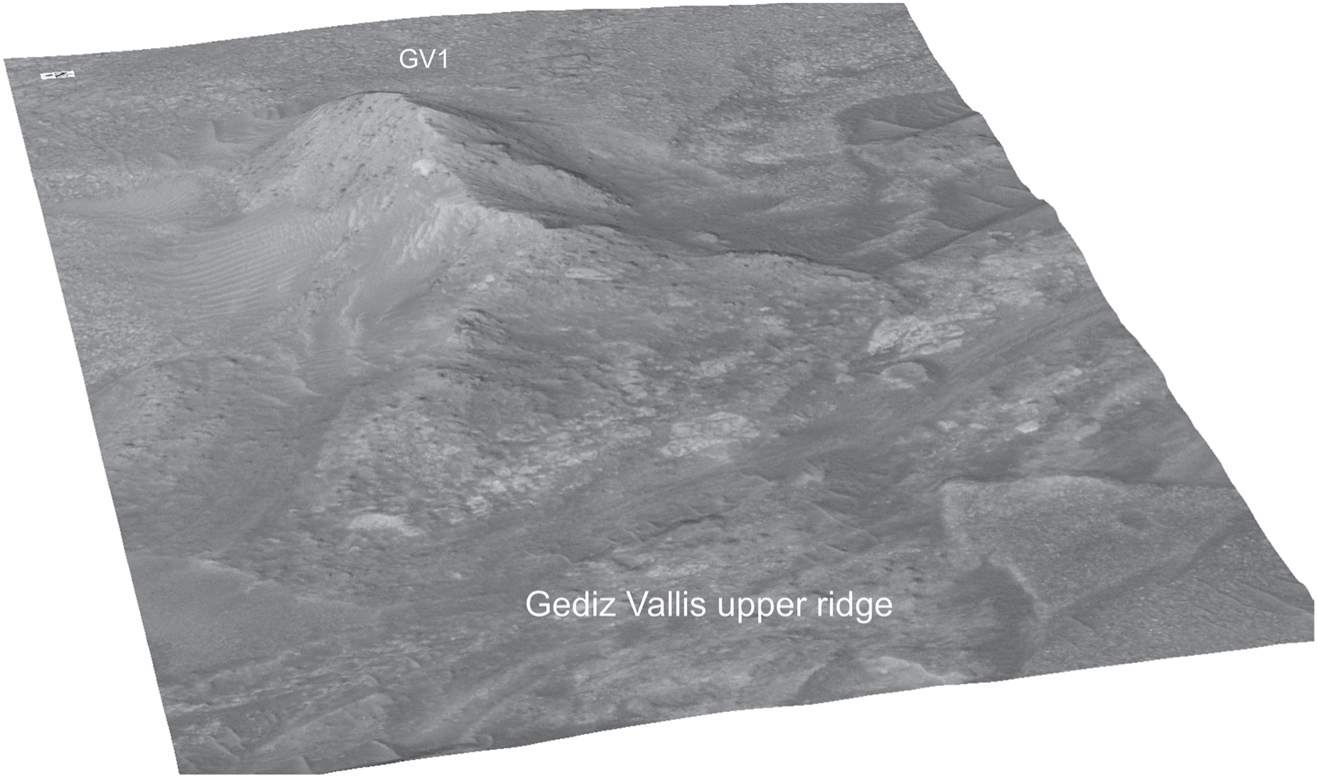
Perspective view of debris ridge GV1 on the eastern Gediz Vallis wall. The ridge is ∼100 m in length, varies in width, and rises ∼6 m in height above the Gediz Vallis floor. The distal end of the ridge, which merges with the Gediz Vallis upper ridge, exposes debris with boulders up to 20 m across.

A single 15 m high, 100 m long debris ridge (GV1, Table 2) extends down the lower portion of the eastern canyon wall where it meets the upper Gediz Vallis channel and ridge deposits (Figures 4, 5, 6). The ridge material consists of blocky, poorly sorted debris similar to the deposits contained within the upper Gediz Vallis ridge proper. The downslope portion of GV1 is truncated where it meets the upper Gediz Vallis channel and ridge. At this location, the debris deposits within the channel exhibit an irregular and subdued topography, in contrast to over‐filling the channel ∼200 m down canyon.

**Table 2:** Debris Deposit Morphometry

Debris ID	Length (m)	Approximate mean width of deposit (m)	Mean slope (degrees)	Surface gradient upslope of scarp or deposit (degrees)	Depth of failure if scarp visible in DEM (m)	Lateral ridges present	Deposited within a chute
SV1	1090	200	13	7	5	yes	yes
SV2	980	180	16	12	4	yes	
SV3	245	100	24	27		yes	yes
SV4	465	20	20	21			
SV5	745	25	19	18			yes
SV6	605	65	21	14			
SV7	550	35	19	21			
SV8	475	30	16	15			
SV9	540	15	15	19			yes
SV10	210	20	25	25			
SV11	740	55	19	13			yes
SV12	760	170	13	12		yes	
SV13	365	55	32	19		yes	
GV1	100	55	10	18			

## 4 Morphologic Characteristics of Sakarya Vallis

**Figure 7:**
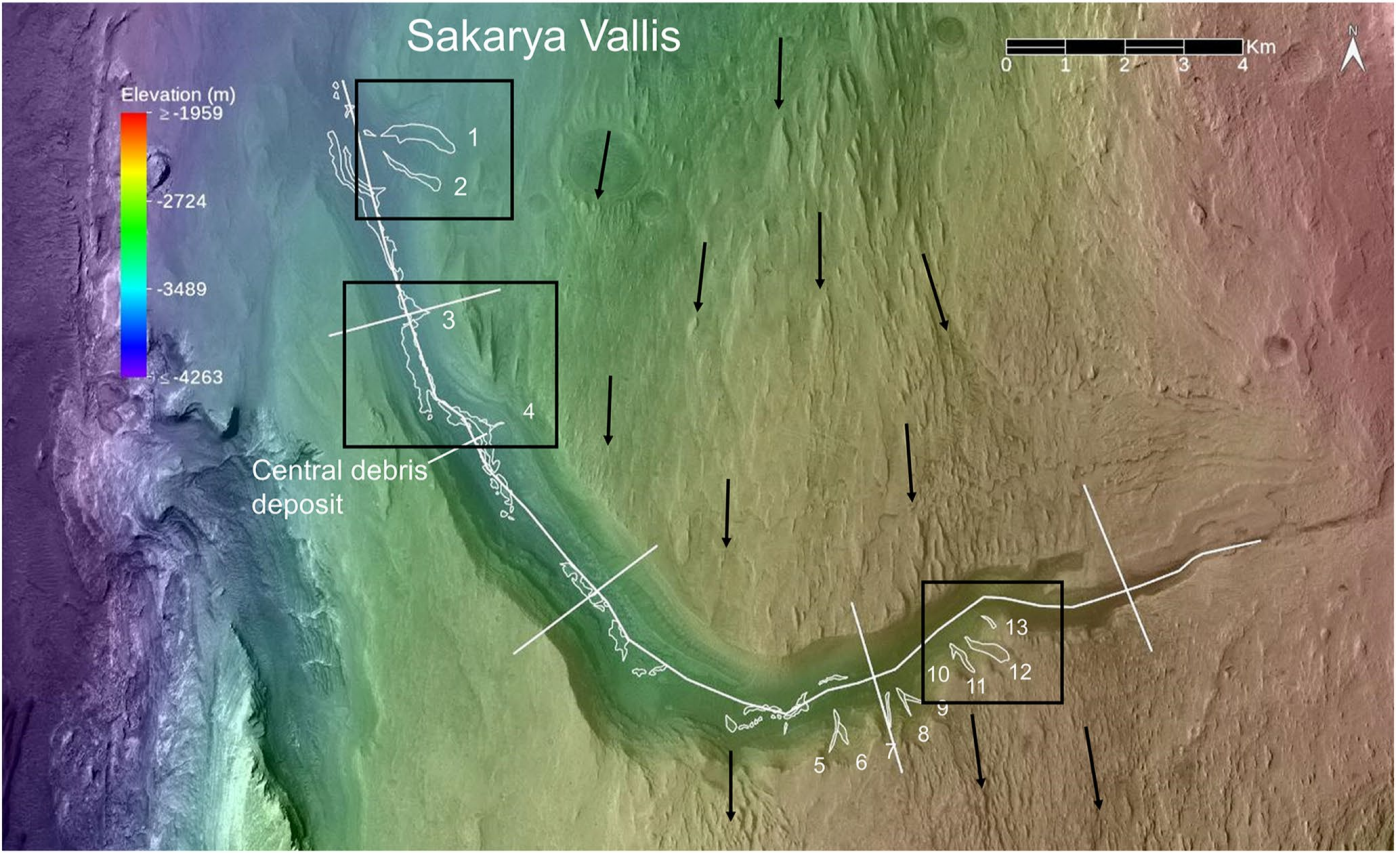
CTX‐based image mosaic covering Sakarya Vallis and adjacent plateaus overlain with color‐coded High Resolution Stereo Camera‐based elevation data (Table 1). White lines delineate the locations for the topographic profiles shown in Figure 8. Debris deposits are labeled within the canyon walls and the floor of Sakarya Vallis (referred to in the text as SV1, SV2, etc. and delineated by numbers only to avoid clutter). Black arrows show inferred wind directions that carved the yardangs on the plateaus. Middle black box delineates the location of the HiRISE data shown in Figure 9, northern one in Figure 11, and southernmost one in Figure 16.

**Figure 8:**
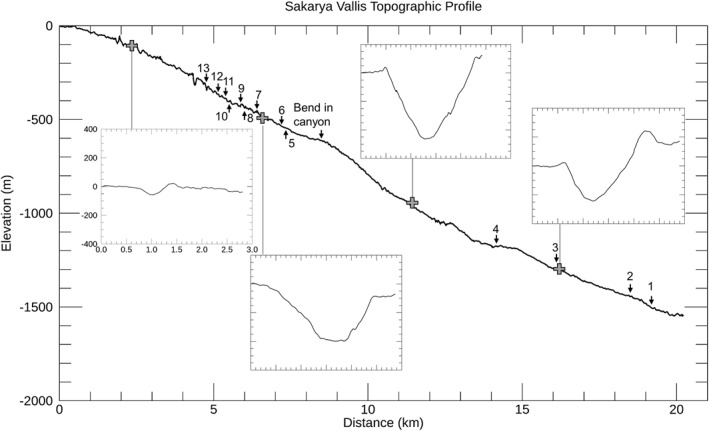
CTX‐based topographic profile along the center of Sakarya Vallis, with cross canyon profiles delineated for key regions. The along canyon profile has a 5.5 vertical exaggeration. Each of the cross‐canyon profiles extends from southern to the northern sides of the canyon and each has a 3.0 vertical exaggeration. The same widths and elevation ranges are shown for each cross‐canyon plot. Locations of the wall debris deposits are labeled, as well as the location of the major bend in the canyon.

Sakarya Vallis is ∼20 km long, with ∼2 km maximum width, and a maximum depth of ∼350 m (Figures 7 and 8). The canyon floor has a slope with a mean value of ∼5°. A nearly 90° bend in orientation from southwest to northwest is evident ∼9 km from the upper‐most portion of the canyon. The canyon's vertical cross section shows progressively increasing width and depth down canyon. The uppermost portion of the canyon is narrow, widening within ∼6 km from its upper reaches to a broader segment (Figure 7). This transition coincides with the spectral detection of sulphate‐bearing strata on the canyon walls and floor, in addition to the surrounding plateaus (Sheppard et al., 2020). We term the upper canyon as the portion located from the initiation of the canyon to the ∼90° bend. The lower canyon extends from the bend to the distal end where the canyon opens onto the plains surrounding Mount Sharp.

The upper canyon is oriented at ∼90° relative to north‐south oriented ridges cut into the surrounding plateau bedrock (Figure 7). In addition, the southern wall at this location exhibits a series of ravines, merging with north‐south oriented ridges on the southern plateau (Figure 7). Wall slopes for the ravines are typically less than ∼20°. In contrast, the northern wall at this location does not exhibit side canyons and exhibits wall slope values ranging up to ∼40°–50°. A portion of the lower canyon northern wall merges with broad, east trending side canyons, whereas most other wall segments in the lower canyon are continuous, lack evidence for side canyons, and have steep slopes with magnitudes similar to those for on the upper canyon northern walls. The walls of the major side canyon in the lower canyon region have slopes less than 20°.

**Figure 9:**
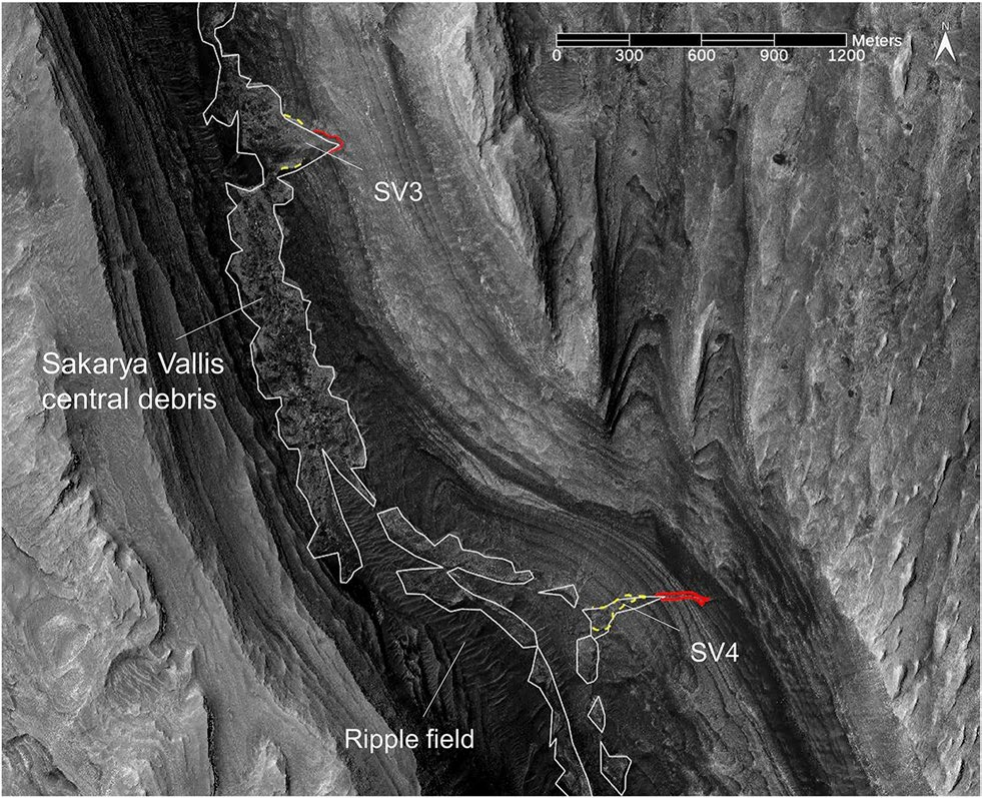
HiRISE‐based view of the central debris deposits on the floor of Sakarya Vallis. Two of the labeled wall debris deposits are also visible. The upper scarps associated with SV3 and SV4 are outlined in red, and the borders of the debris deposits are outlined in dashed yellow. Ripple fields cover portions of the central debris in the southern half of the image. Based on HiRISE orthorectified image and digital elevation models data from PSP_006855_1750 and PSP_007501_1750. Same data used for Figures 10, 11, 12, 13, 14, 15.

**Figure 10:**
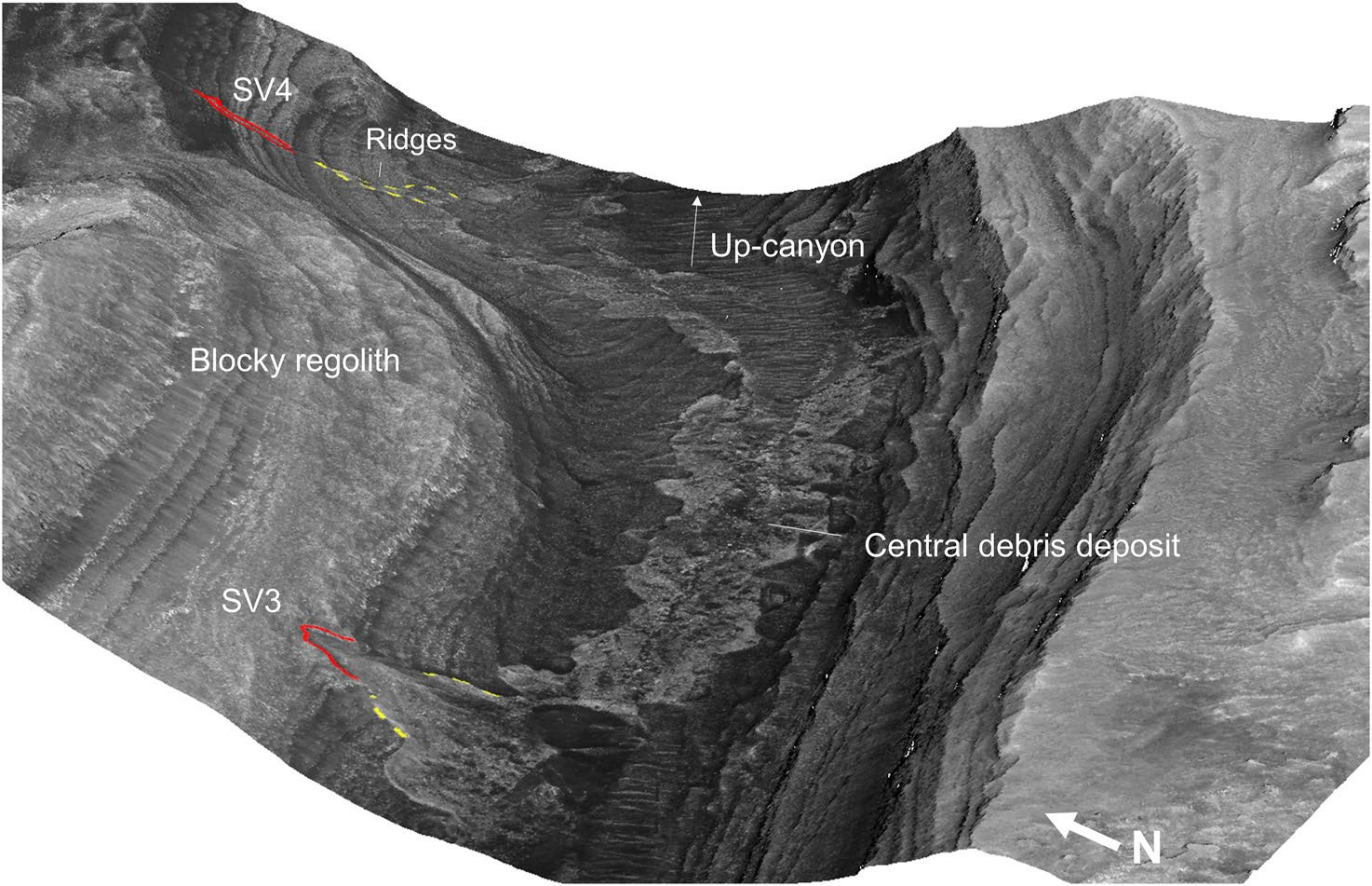
Perspective view of the region shown in Figure 9. For scale reference, the width of the central debris deposit where labeled is ∼250 m. The wall debris deposits, SV3 and SV4 merge with the central debris deposit located on the canyon floor. The head scarps of the two wall deposits are outlined in red and their lateral borders are outlined in dashed yellow.

As compared to Gediz Vallis, the floor of Sakarya Vallis is in many places covered by wind‐blown sand sheets and ripple fields that preclude areally extensive examination of canyon floor deposits, particularly in the upper canyon. A ∼3 km portion of the lower canyon floor is well exposed and exhibits a sinuous debris ridge that ranges in width up to ∼300 m, rising several meters above the surrounding floor rocks (Figures 9 and 10). The ridge exhibits blocky, poorly sorted debris, with boulders up to ∼10 m across. Debris ridges are also evident further down‐canyon. In some locations, channels cut into these deposits, and in other areas debris fills the channels. Most of the undulations of the floor debris are located immediately downhill from arcuate escarpments cut into the canyon lower wall strata (visible in Figures 9 and 10). In other locations, the floor deposits merge with debris ridges and chute‐filling debris deposits that extend down the canyon walls. In contrast to Gediz Vallis, with its upper ridge and massive lower ridge, Sakarya Vallis does not show a massive debris ridge where it terminates on the plains surrounding Mount Sharp. Only a modest amount of material is evident at the canyon's distal end.

As part of our study, we mapped and characterized thirteen well‐exposed debris deposits and associated features on the walls of Sakarya Vallis (Table 2, Figures 7, 8, 9, 10, 11, 12, 13, 14, 15, 16) as follows:

**Figure 11:**
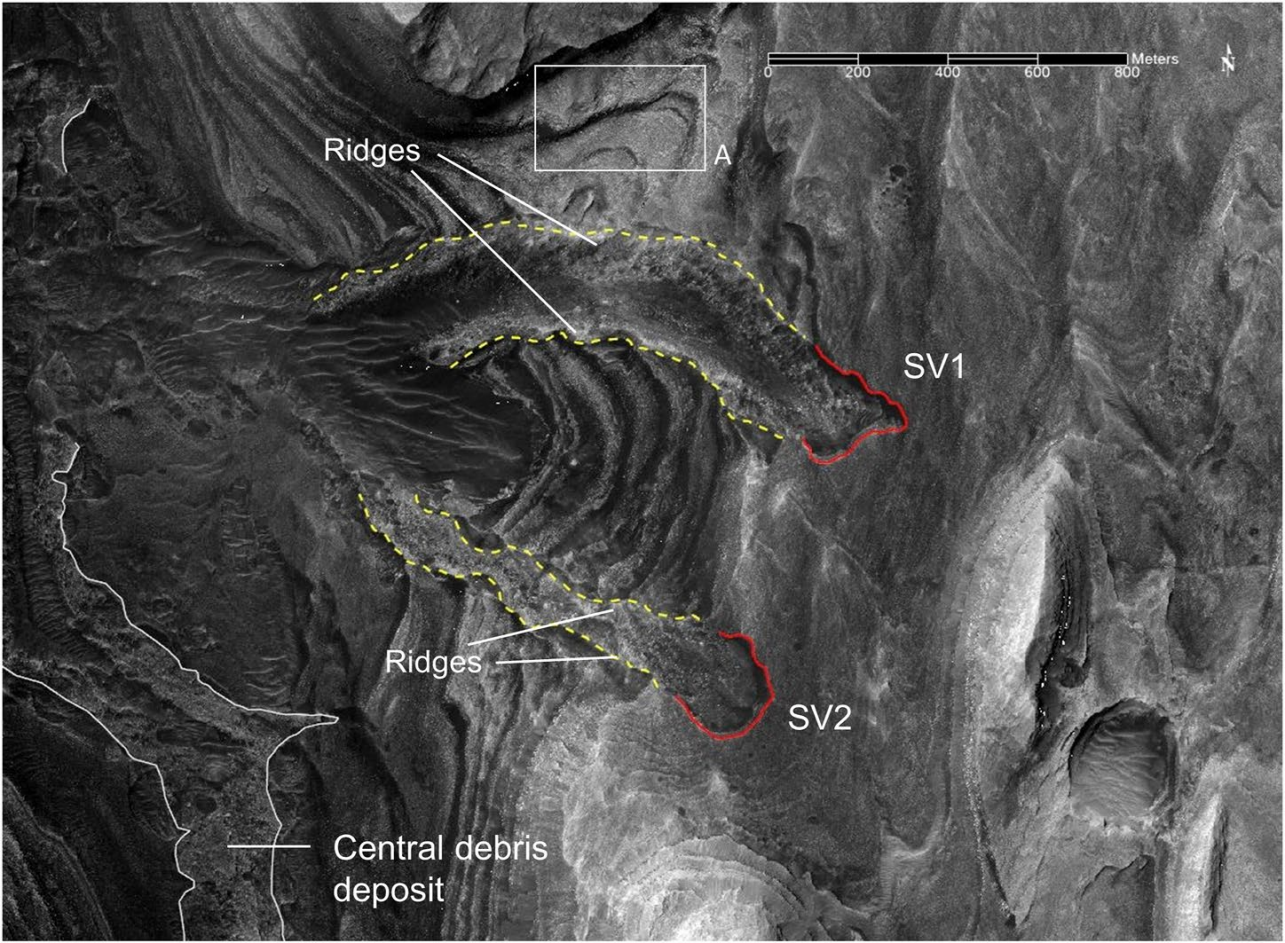
A portion of HiRISE image with mapped chutes, debris, and lateral ridges for SV1 and SV2 in Sakarya Vallis. Head scarps outlined in red, debris deposit borders outlined in dashed yellow. Lateral ridges in the debris deposits are labeled. The white box covers the region with an extensive disaggregated bedrock cover shown in Figure 12.

**Figure 12:**
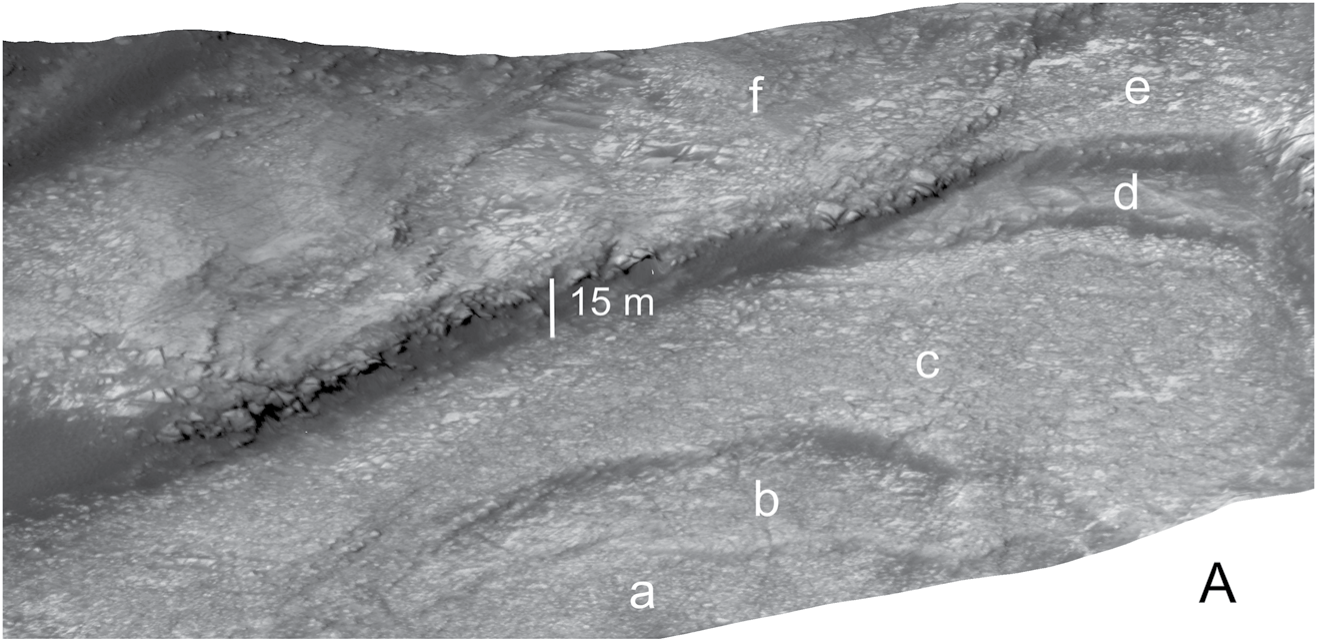
Perspective view looking to the northeast of a portion of the valley shown in Figure 11 Letters a through f delineate disaggregated bedrock associated with different exposed strata. The vertical line corresponds to 15 m of relief. The surface shown for area “f” was used to compute a blocky regolith bulk density as described in the text.

**Figure 13:**
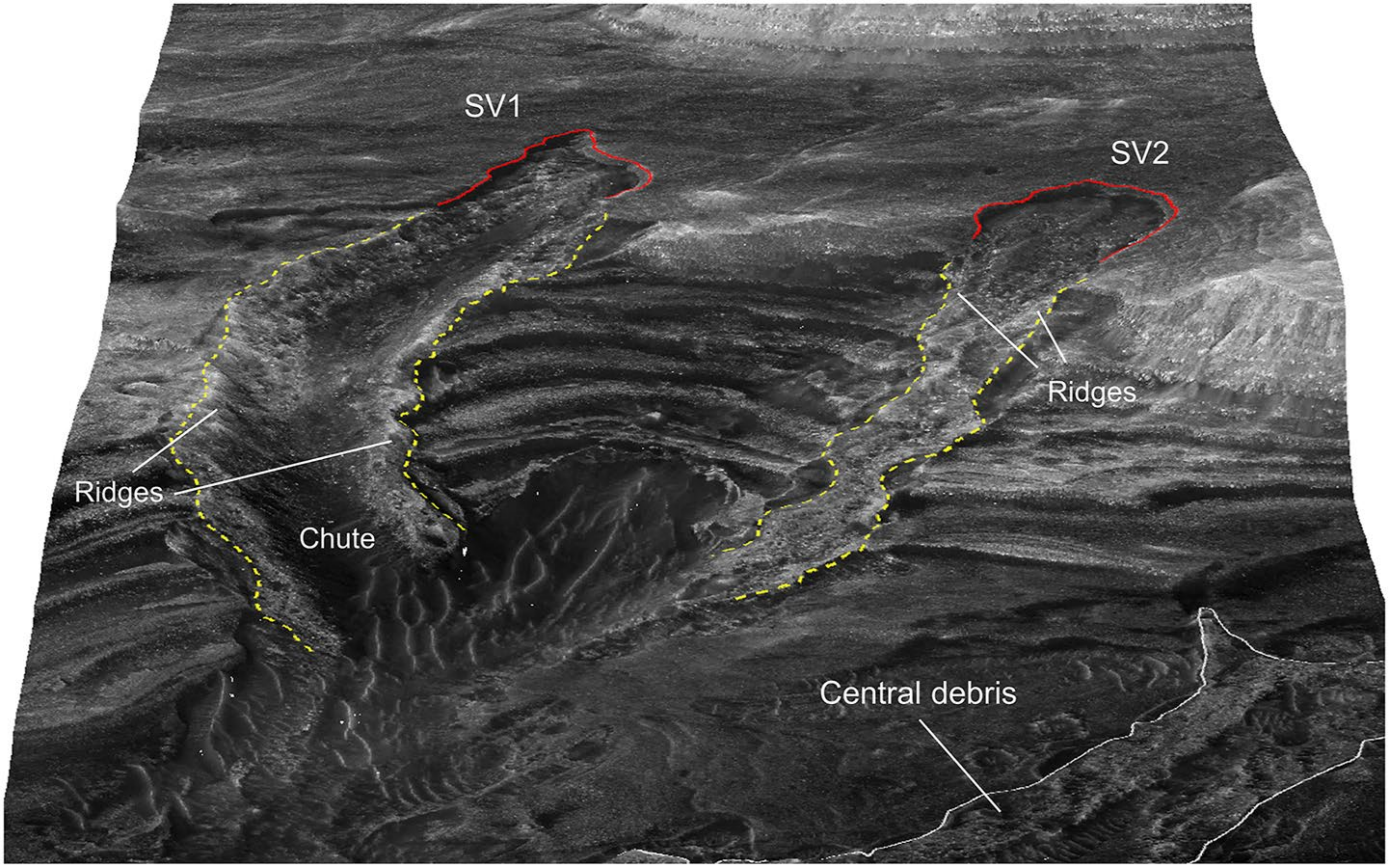
HiRISE‐based perspective view looking toward the east of the area covering SV1 and SV2. Head scarps outlined in red, debris deposit borders outlined in dashed yellow. Lateral ridges in the debris deposits are labeled. Note the central debris shown in the lower right of the figure along the floor of the canyon, along with the disaggregated bedrock surrounding the debris deposits. For scale reference, the width of SV1 is ∼200 m.

**Figure 14:**
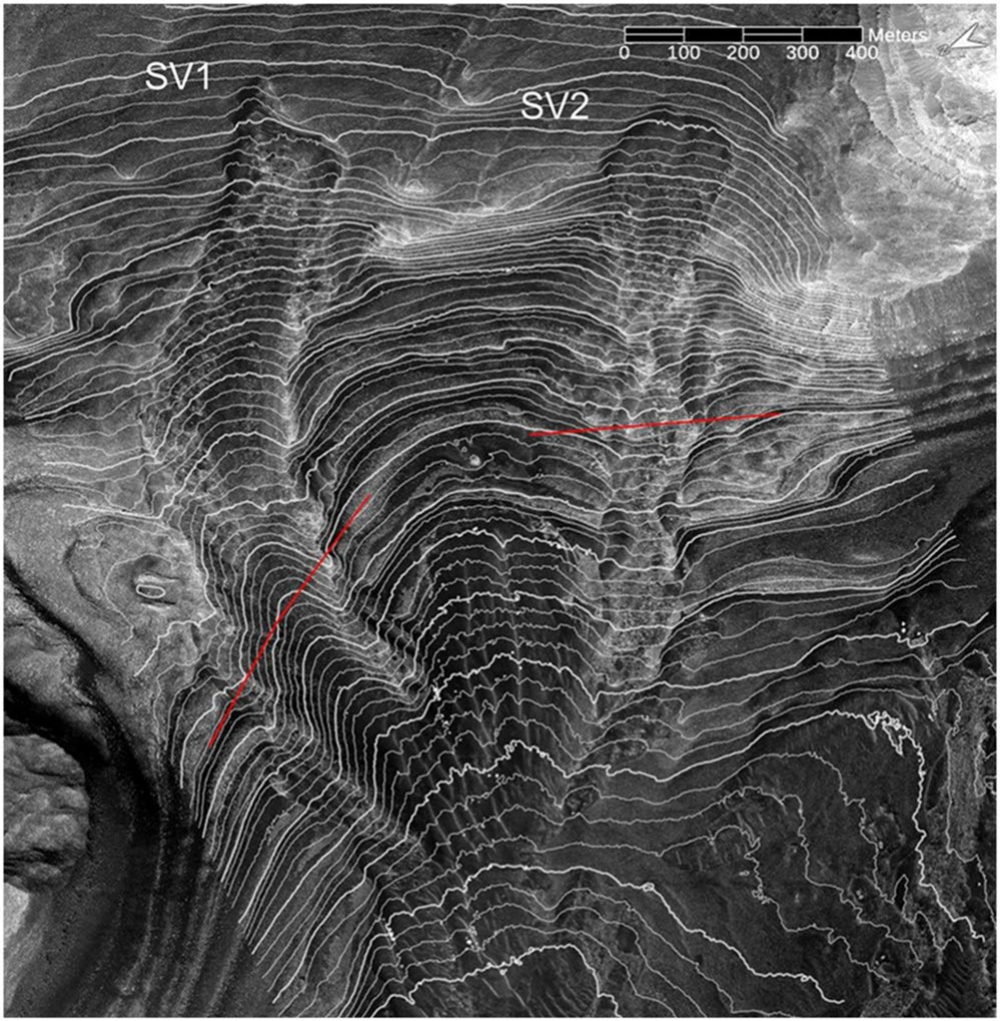
HiRISE‐based view of SV1 and SV2 with 5 m contours overlain in white. Twenty m contours are shown with a thicker white line. The elevation range of the contour lines is from −3,260 m to −3,740 m. The topographic profiles indicated by the two red lines are shown in Figure 15. North is approximately towards the left.

**Figure 15:**
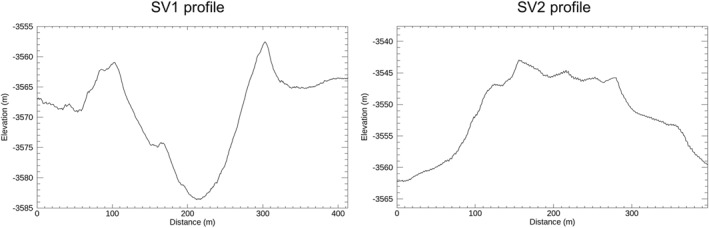
HiRISE digital elevation models‐based topographic profiles crosscutting SV1 (left) and SV2 (right). The locations of the profiles are shown in Figure 14. Both profiles have an 8 times vertical exaggeration. Note that the SV2 profile, in particular, shows that this deposit and associated features are now perched above surrounding bedrock exposures.

**Figure 16:**
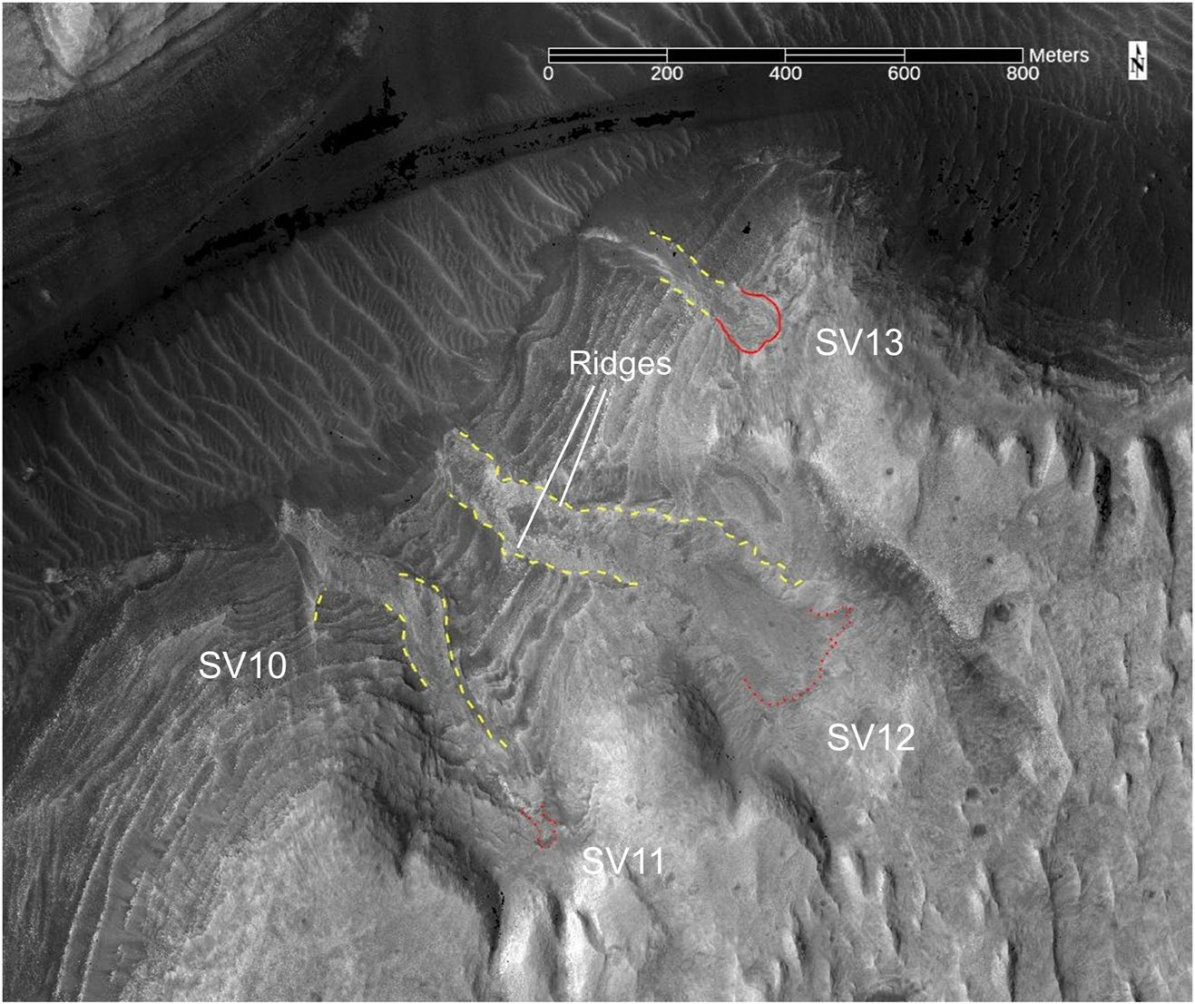
A portion of HiRISE image covering SV10 to SV13. These debris deposits are on the southern wall within side canyon ravines cut into the plateau. Same labeling scheme as used in Figure 11. Based on HiRISE orthorectified and digital elevation models data ESP_012340_1750, ESP_012195_1750. Also used for Figure 17.

Debris deposit SV1 is located on a lower canyon side canyon. It is ∼1,090 m long and ∼200 m wide, with a mean downhill slope of ∼13°. SV 1 is contained in a chute cut into the wall strata. The chute floor has a maximum depth of ∼20 m relative to surrounding wall rocks, and has lateral debris‐laden ridges that stand up to several meters above the surrounding wall rocks (Figures 11, 13, 14, 15). The chute exhibits an arcuate head scarp that has a relief ∼5 m relative to the wall rocks located immediately uphill of the scarp. The lower chute has less debris fill and exhibits a relatively smooth‐looking floor as compared to the upper chute. The deposit within the upper chute consists of blocky, poorly sorted debris. Wind‐blown sand covers the distal end of SV1 where the debris meets the canyon floor, although windows through the sand cover show that the deposits merge with debris deposits on the canyon floor. These windows also show that the distal end of SV1 curves and tends to align with the down‐canyon direction. The strata north of SV1 provide a good exposure of the sulphate‐bearing, disaggregated bedrock that dominate the walls of the canyon (Figures 11 and 12).

SV2 is contained within a chute bound by lateral ridges, and begins in an arcuate‐shaped head scarp exhibiting ∼4 m of relief relative to the wall strata immediately uphill (Figures 11, 13 and 14). Downslope, chute‐bounding lateral debris ridges rise several meters above the surrounding wall rocks, as do portions of the debris deposit proper. The chute‐filled debris deposit is ∼980 m long and ∼180 m wide, with a mean slope of ∼16°. Debris in the chute forms a series of irregular internal ridges aligned with the down‐slope chute direction (Figures 14 and 15). Wind‐blown sand covers the distal end of SV2 where the debris meets the canyon floor, although windows through the sand cover show that the deposits merge with debris deposits on the canyon floor.

SV3 and SV4, located on the northern canyon wall, begin in shallow head scarps, and transition downslope to debris ridges (Figures 9 and 10). The SV3 ridge begins as a narrow feature, broadening in width down‐slope and connecting to floor debris deposits. Subdued lateral ridges bound the lower portion of SV3. SV4 also begins in a narrow head scarp, transitions to a v‐shaped ridge, and connects to the central floor debris deposits.

**Figure 17:**
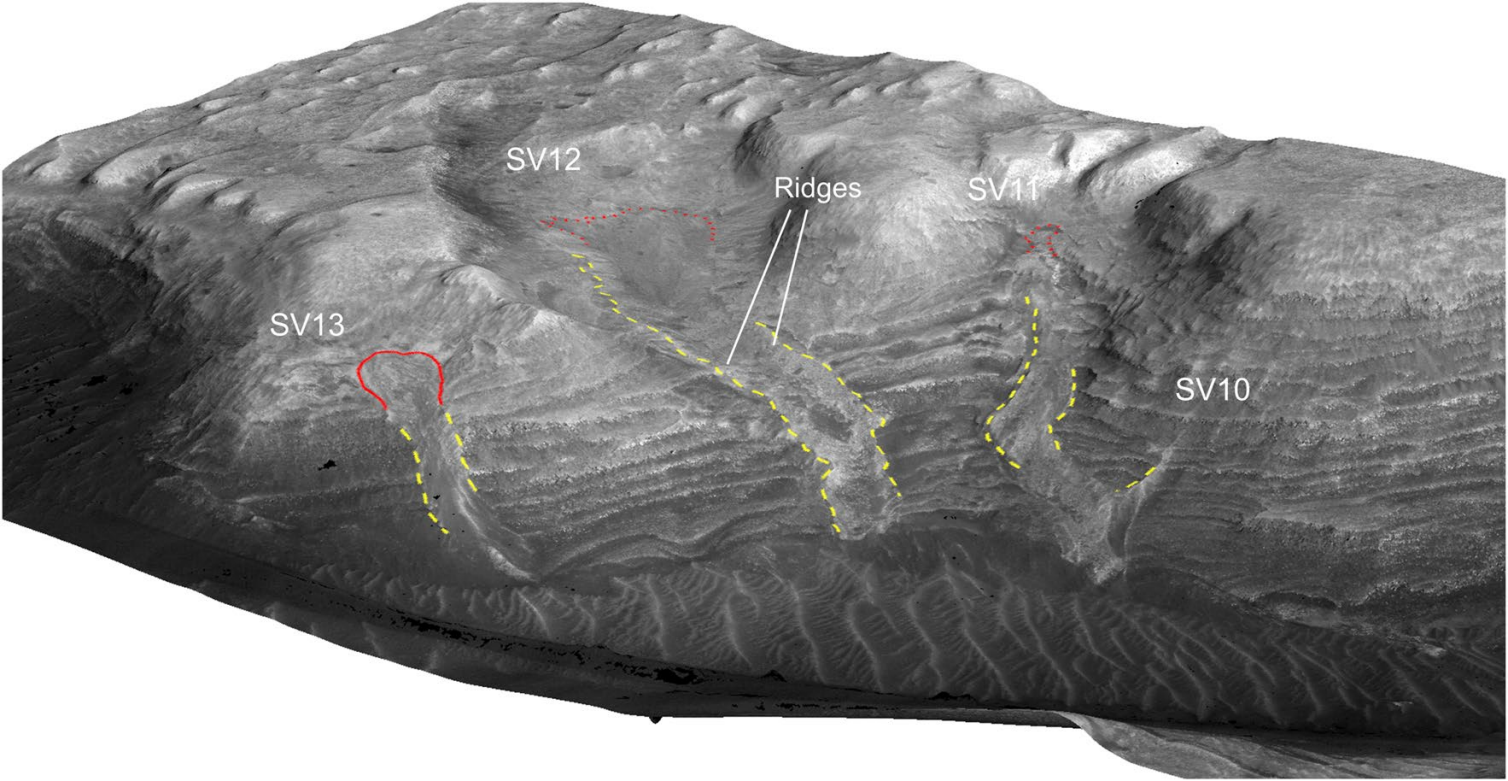
HiRISE‐based perspective view covering SV10 to SV13. The debris deposits are covered by basaltic sands where they meet the canyon floor. For reference, the width of SV13 is ∼55 m.

SV10, SV11, SV12, and SV13 are located in the upper portion of the canyon in side ravines extending uphill into the southern plateau (Figures 7, 16 and 17). SV13 begins at a ∼90 m wide head scarp, followed by debris that extends downhill for ∼365 m, with a mean slope of ∼32°. The upper 100 m exhibits a subtle trough cut ∼1 m into bedrock, whereas the lower portion is a series of debris ridges standing several meters above the surrounding bedrock.

The head of SV12 lies in a broad, relatively gently sloped valley, and exhibits a poorly defined head scarp (denoted by the dashed red line in Figures 16 and 17). The upper portion of this deposit exhibits a debris‐filled shallow chute that is ∼170 m wide, with a mean slope of ∼13°. The lower portion exhibits a shallow debris‐filled chute containing a debris ridge. The upper portion of SV11 also lacks a well‐defined head scarp (candidate head scarp is outlined in Figures 16 and 17) and is a ∼55 m wide debris ridge contained within a shallow chute. It merges downslope with SV10, a narrow debris ridge that stands a few meters above the surrounding bedrock exposures. SV11 is ∼740 m in length, with a mean slope of ∼19°. All of the deposits are blocky and poorly sorted where visible. Wind‐blown sands cover the deposits where they meet the canyon floor.

SV5 to SV9 are also located within ravines cut into the upper canyon's southern wall, and have similar morphologies as SV11. These debris deposits are 15–65 m wide and up to ∼745 m long. Mean slopes are comparable to the ravine debris deposits discussed in the previous paragraph. These blocky, poorly sorted deposits are ridges rising a few meters above the surrounding bedrock. They do not exhibit even poorly defined head scarps and lateral ridges are not evident. Wind‐blown sands cover these debris deposits where they meet the canyon floor.

## 5 Generation of Blocky Regolith From Sulphate‐Bearing Strata

In this section we consider the mineralogy of the sulphate‐bearing strata exposed on the walls of Gediz and Sakarya Valles and implications for production of blocky regolith by partial dissolution of the sulphate‐bearing rocks. Specifically, CRISM data covering Mount Sharp has shown that magnesium‐bearing, hydrated sulphate‐bearing rocks dominate the spectral signatures for the stratigraphic section between the upper light toned strata and the lower portions of Mount Sharp dominated by Murray formation outcrops (Milliken et al., 2010; Sheppard et al., 2020; Thomson et al., 2011). These rocks are likely similar to the sulphate‐bearing Burns formation rocks characterized by the Opportunity Rover at Meridiani Planum. Opportunity Mini‐TES data indicate that the Burns formation rocks are composed of ∼40% sulphate minerals, mainly magnesium and calcium‐bearing phases, with ∼60% composed of aluminum‐rich amorphous silica, plagioclase, nontronite, hematite, and minor phases (Glotch et al., 2006).

**Figure 18:**
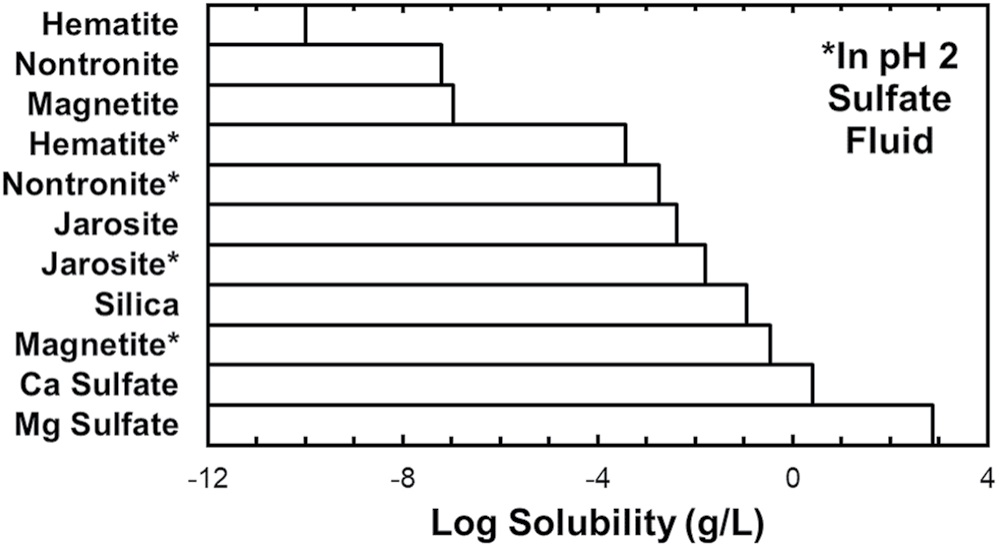
Histogram showing the log solubility for relevant secondary minerals that have been identified in Burns formation rocks in Meridiani Planum, a mineral analog for the sulphate‐bearing strata on Mount Sharp. Note the high solubility of magnesium sulfates, followed by calcium sulfates. Minerals that are more soluble in low pH fluids were calculated with pH of 2 to show that even then they are much less soluble than the sulphate minerals.

Magnesium sulphate minerals have orders of magnitude greater relative solubility as compared to most other minerals, including oxides and silicates, as shown in Figure 18. To evaluate in more detail the hypothesis of enhanced aqueous disaggregation of the sulphate‐bearing strata to produce blocky regolith we employed geochemical modeling assuming a temperature of 25°C to estimate the fraction of Mg and Ca sulfates that would dissolve in a water‐infiltrating environment. The temperature was chosen based on existence of good thermodynamic data. Additional exploratory calculations at slightly lower temperatures showed that solubilities did not change appreciably. We used the Mini‐TES based mineralogy of the Burns formation as an analog for the sulphate‐bearing rocks that underlie Mount Sharp. Assuming a Burns formation estimated porosity of 4.5% (Nahm & Shultz, 2007), and saturated conditions, we find that ∼2% by volume of the magnesium and calcium sulfates would dissolve in the presence of infiltrating moisture from precipitation or snowmelt. The porosity used is a minimum value for most sandstones, making this a conservative estimate. This could lead to disaggregation of bedrock to generate a blocky regolith, for example, as infiltrating water preferentially moved along permeable bedding planes and joints within the rocks.

We also note that the stable hydrated magnesium phases depend on moisture content, with large volume changes as moister conditions lead to more hydrated phases. For example, conversion of kieserite (MgSO_4_·H_2_O) to starkeyite (MgSO_4_·4H_2_O) produces a 69.4% volume increase because three additional H_2_O molecules are added to the unit cell. If the pore water content varied due to changing climatic conditions then the consequent volume changes could also enhance disaggregation.

Another special property of sulphate‐bearing strata, based on comparisons to the Burns formation rocks, may be their relatively low strength. Both the Spirit and Opportunity rovers deployed their Rock Abrasion Tools to grind a few millimeters into rock targets to remove dust and other coatings before making contact science measurements (Gorevan et al., 2003). The olivine‐bearing basalts on the Gusev plains, combined with various silicate‐dominated rocks within the Columbia Hills, completely wore down the grinding teeth before the end of the mission (Arvidson et al., 2010). The Adirondack class basaltic rocks on the plains had specific grind energies ranging from 30 to 50 J/mm^3^ retrieved from Rock Abrasion Tool (RAT) telemetry that are equivalent to uniaxial compressive strengths of ∼70–130 MPa (Thomson et al., 2013). On the other hand, Opportunity RAT data for grinds into sulphate‐rich Burns formation rocks free of the very hard hematitic concretions averaged ∼1 J/mm^3^ (Arvidson et al., 2004), equivalent to much lower compressive strengths than found for most rocks at Gusev Crater, with the exception of the sulphate‐bearing Peace sandstone (Thomson et al., 2013). Of course, a grind depth of a few millimeters may not be representative of the strength of deeper portions of the rocks and sulphate‐bearing strata found on Mount Sharp may not have the same strength characteristics as Burns formation rocks.

We hypothesize that the relatively high solubility of sulphate minerals, large volume changes due changes to varying moisture contents, and perhaps a relatively low strength expected for sulphate‐bearing rocks that underlie Mount Sharp, would all contribute to the production of disaggregated bedrock. These inferences are certainly consistent with the appearance of the bedrock shown in Figure 12, material on the canyon walls that would be preconditioned to fail as landslides.

## 6 Conceptual Models for Producing the Debris Deposits

In this section, we discuss possible origins for the debris deposits and associated features, for example, chutes, lateral ridges, head scarps, and the poorly sorted nature of the debris. None of the deposits, associated features, and surrounding bedrock exposures has escaped erosion by wind, given that many are debris ridges and stand high above the surrounding wall rocks (e.g., GV1). They thus fall into the category of inverted channels or ridges found in many other places on Mars (e.g., Pain et al., 2007; Williams et al., 2007; Zaki et al., 2021).

### 6.1 Glacial Processes

The lower slopes of Mount Sharp explored by Curiosity do not show any conclusive evidence for the effects of glaciation on the landscape. Curiosity's imaging data acquired for the upper portion of Mount Sharp likewise do not show any evidence for the effects of glacial activity. Mount Sharp lacks evidence for bedrock scour marks, rock polish, lateral or terminal moraines, eskers, roche moutonnée, drumlins, landscape features such as hanging U‐shaped valleys, horns, and arêtes.

One could argue that GV1 is an eroded vestige of a one much more extensive moraine. The orientation and shape are consistent with that of a terminal moraine, but the absence of any lateral moraines makes this origin less likely. It is not obvious that wind erosion would preferentially erode only lateral moraines. To be a recessional moraine, with no trace of lateral moraines, would require that a much more extensive glacier existed, with the equilibrium line downstream of the ridge deposition. In that case, the ridge deposit would record a short‐lived recessional feature and likely not be one that stands, even after wind erosion, ∼15 m above the surrounding bedrock exposures.

The lack of glacial landforms also argues against the hypothesis that the debris deposits in both Gediz and Sakarya Valles are eskers. Rather, the relatively well‐preserved debris deposits in Sakarya Vallis argue for a downslope mobilization and transport of poorly sorted debris without associated lateral, terminal, or recessional moraines that would have been deposited by glaciers.

### 6.2 Downhill Creep and Dry Landslides

Another hypothesis that needs exploration is whether the debris deposits could simply be due to a combination of dry landslides and extensive downhill creep into canyons without the enabling role of water. Creep of the surface mantle on hillslopes is typically advanced by internal deformation under a state of shear (mostly likely in clay rich materials) or through some form of dilational disturbance (e.g., freeze thaw and heating and cooling). Raveling can occur by individual blocks that eventually are released from bedrock through progressive fracture propagation and by physical/chemical reactions described above. Accumulation through time can lead to extensive blocky regolith cover. Presently there are local talus aprons with traces of individual block tracks still visible in some steep canyon walls in Gale crater.

Dry landslides (rockfall; e.g., Okura et al., 2000) are judged to be unlikely here because of the relatively low wall slopes associated with many of the debris deposits (Table 2). The slope values are far below angles of repose for failure of regolith, even for materials with low cohesive strengths (e.g., Selby, 1993 and Table 2). Dry landslides do remain a possibility for debris deposits associated with steeper slopes (Table 2).

We note that acoustic fluidization (Collins & Melosh, 2003) has been hypothesized as a mechanism for long landslide runout lengths. This mechanism presumes an initial fall height of a mass that transfers a portion of its kinetic energy to internal acoustic vibrational energy. The debris deposits summarized in Table 2 are not associated with significant free fall heights, making the role of acoustic fluidization unlikely.

Finally, regolith creep and dry landslides could not transport the coarse debris on the modest valley floor slopes of Gediz Vallis and Sakarya Vallis, nor cut channels. For Gediz Vallis, in particular, these processes are unlikely to have formed either the channel on the floor that is over‐filled with debris, or the massive lower Gediz Vallis ridge. A great deal of material has been transported down the ∼10° canyon floor to the terminus to merge with the lower Gediz Vallis ridge. The slope is judged to be too low for this process to operate under dry only conditions.

### 6.3 Fluvial Activity, Landslides, and Debris Flows

We argue that the observations of the debris deposits and associated features within Gediz and Sakarya Valles are best explained by a late stage evolution of the canyons in which the presence of surface and ground water enabled the mobilization and downslope transport of extensive volumes of blocky debris. The evidence to support this assertion is largely contained in the relatively well‐preserved deposits and features in Sakarya Vallis. We use these deposits and features to develop a conceptual model, which we use to formulate the sequence of events that led to the present configuration of debris deposits and associated features in Gediz Vallis. We note that, as on Earth, many landslides are associated with precipitation and associated groundwater flow, providing the triggering mechanisms to initiate shear failures and landslides by reduction of effective stresses due to increased pore water contents within the incipient failure mass (e.g., Dietrich et al., 2001).

Late‐stage landslide features with similar morphologies to features in Sakarya Vallis have been identified in other locations on Mars, including examples hypothesized to start as landslides that developed into debris flows due to the presence of groundwater and melting subsurface ice (e.g., Guimpier et al., 2021; Lucchitta, 1979, 1987; Quantin et al., 2004). In our case, SV1 and SV2 within Sakarya Vallis exhibit arcuate shaped head scarps, chutes extending downslope partially or completely filled with blocky, poorly sorted debris, and chute‐bounding lateral debris ridges. These features are also located on slopes less than ∼20°. These properties are all consistent with a series of slope failures as landslides that mobilized as debris flows (e.g., Iverson, 1997; Kaitna et al., 2016). The debris moving downslope would have carved the chutes into wall rocks. In fact, physical modeling (Hsu et al., 2011) and observations of terrestrial debris flow deposits (Stock & Dietrich, 2003) demonstrate the ability of debris flows to erode into underlying materials. Downslope transport would have produced lateral stresses in the debris flows that pushed material outward from the faster moving flow center, thereby generating debris‐laden ridges (levees) in the same manner as occurs in terrestrial debris flows (Iverson, 1997).

Local precipitation and ground water infiltration, melting subsurface ice, and ground water transport from higher up Mount Sharp are all viable mechanisms of triggering failures. In terrestrial cases, debris flows develop from landslides destabilized by elevated pore water pressure. When the mass failure occurs, water mixes with poorly sorted debris able to transport sediment that can carve chutes, produce levees, and transport poorly sorted materials at relatively low slopes (e.g., Iverson, 1997; Kaitna et al., 2016). We envision a similar role of water for the debris deposits discussed in the previous two paragraphs, that is, SV1 and SV2, although neither feature is consistent with a single event, nor escaped some erosional degradation.

We also note that there is no evidence of river channels entering the headscarps of SV1 and SV2, suggesting the importance of ground water transport to the landslide sites. On the other hand, wind erosion may have removed upslope channel topography, and erased evidence of fluvial incision downslope.

The remainder of the debris deposits within Sakarya Vallis lack one or more key features that allow these deposits to be placed in the same conceptual model as debris flows enabled by the presence of water. So little of the original topography is preserved at SV3 and SV4, it is difficult to assess the possible mechanisms of transport and deposition that lead to these deposits. Likewise, debris deposits SV5–SV9 and GV1 lack features that allow an interpretation of the role of water in landslide failures and debris flows. We interpret the ridge‐only morphology of these deposits to be a consequence of differential wind erosion, that is, they are analogous to fluvial “inverted channels” that have been mapped elsewhere on Mars (Pain et al., 2007; Williams et al., 2007). That is, these debris deposits have suffered less erosion than the surrounding wall rocks, leaving the deposits as ridges standing above the surrounding rocks. Some of the deposits may have accumulated over time from upslope raveling, perhaps driven by moisture sensitive regolith production and block release in the sulphate bearing bedrock, as described above.

The conceptual model for slope failure and debris flow generation described for SV1 and SV2 could also apply to debris deposits SV11 to SV13 located in the upper canyon ravines. However, these deposits also lack, or have poorly defined, diagnostic features and the potential role of water cannot be judged. Both SV12 and SV11 lack distinct head scarps (Figures 16 and 17). The outlined features may be degraded head scarps, or they could be the uppermost extent of the debris features that have not yet been removed through erosion. SV12 may have remnants of lateral debris ridges (Figures 16 and 17). However, these are very subdued ridges and could have been formed through differential aeolian erosion after the debris was deposited.

## 7 Exploratory Model for Landslide and Debris Flows

In this section, we evaluate, in an exploratory manner, the amount of water needed to initiate landslides in the blocky regolith on the canyon walls as a function of the shear strength at the failure plane and the slope of the wall. We employ an infinite slope model (i.e., one‐dimensional Mohr‐Coulomb based failure) with groundwater flow as the driving mechanism for failure. The ground surface and failure plane are assumed to be parallel (e.g., Dietrich et al., 2001; Selby, 1993). In our case, the failure plane corresponds to the assumed boundary between blocky regolith and underlying less weathered wall rocks (e.g., Figure 12). Given the extensive post‐formation wind erosion associated with both canyons, and thus the difficulty of estimating the true slopes during the failure periods, we pursue calculations for both a range of properties and slopes.

In the model, failure occurs when the downhill stress component (i.e., shear stress, τ) due to the overlying weight is equal to the combined cohesive and frictional strength (S) at the failure plane. Groundwater entering the blocky regolith elevates the pore pressure, lowers the effective normal stress, and thereby reduces the frictional strength. The model neglects wall stresses, which act to stabilize against failures (e.g., Milledge et al., 2014; Prancevic et al., 2018), making our estimates of water content for failure a minimum. It also assumes that water flow is parallel to the failure plane. With these assumptions, the proportion of the regolith layer saturated at failure is equal to:

(1) $$\frac{h}{z}=\frac{\rho_{s}}{\rho_{w}}\left(1-\frac{\tan\;\theta }{\tan\;\varphi }\right)+c/(\rho_{w}{gz\cos}^{2}\theta \tan\varphi )$$

where:

*z* (L) is the thickness of the blocky regolith above bedrock*; h* (L) is thickness of the saturated zone above the assumed failure plane*; ρ*_*s*_ (M/L^3^) is the regolith bulk density*; ρ*_*w*_ (M/L^3^) is the water density, assumed to have a value of 1,000 kg/m^3^ *; θ*, is the failure plane slope angle*; φ* is the angle of internal friction at the boundary between the blocky regolith and underlying bedrock*; c* (M/LT^2^) is the cohesive strength at the boundary*; g* (L/T^2^) is the gravitational acceleration.

Model assumptions limit the range of *h/z* from 0.0 (dry) to 1.0 (saturated). Values higher than 1.0 suggest that instability under such circumstances would be due to exfiltration gradients elevating the pore pressure beyond that just due to saturation (e.g., Dietrich et al., 2001) or due to flowing surface water (Prancevic et al., 2014). Strong exfiltration gradients can lead to instability and even liquefaction of the material (e.g., Iverson & Major, 1986), and surface water can lower the threshold slope for mass failure (Prancevic et al., 2014).

We used an upper bound for the bulk density of the blocky regolith based on the value for Burns formation consolidated sulphate‐bearing rock estimated to be ∼2.5 g/cm^3^ (Grotzinger et al., 2005). Estimates of bulk densities of Martian regolith are for granular materials worked by lander and rover wheels and scoops (e.g., Moore et al., 1987; Shaw et al., 2009; Sullivan et al., 2011; Knuth et al., 2012; Morgan et al., 2018). These values range from ∼1 to 1.8 g/cm^3^. Measurements for blocky regolith on Mars do not exist. Estimates for terrestrial weathered bedrock range from ∼1.2 to 2.0 g/cm^3^ (Wald et al., 2012). Estimates of terrestrial “broken and blasted rock” range from 1.0 to 2.2 g/cm^3^ (Hoek & Bray, 1999). We also estimated bulk density from HiRISE data covering a typical surface covered with disaggregated bedrock (Figure 12). For the location chosen for analysis, blocks cover 60% by area, with the remaining 40% covered by sands and fine‐grained regolith. Assuming a rock density of 2.5 g/cm^3^ and a sand/regolith density of 1.5 g/cm^3^, we estimated a blocky regolith density of 2.1 g/cm^3^. With regard to cohesive strength, martian granular regolith values are estimated to range from 1 to 5 kPa, and angles of internal friction from 20° to 40°, based on the references quoted in the above paragraph. “Broken and blasted rock” that are deemed to be cohesionless have angles of internal friction ranging from 20° to 40° (Hoek & Bray, 1999).

**Figure 19:**
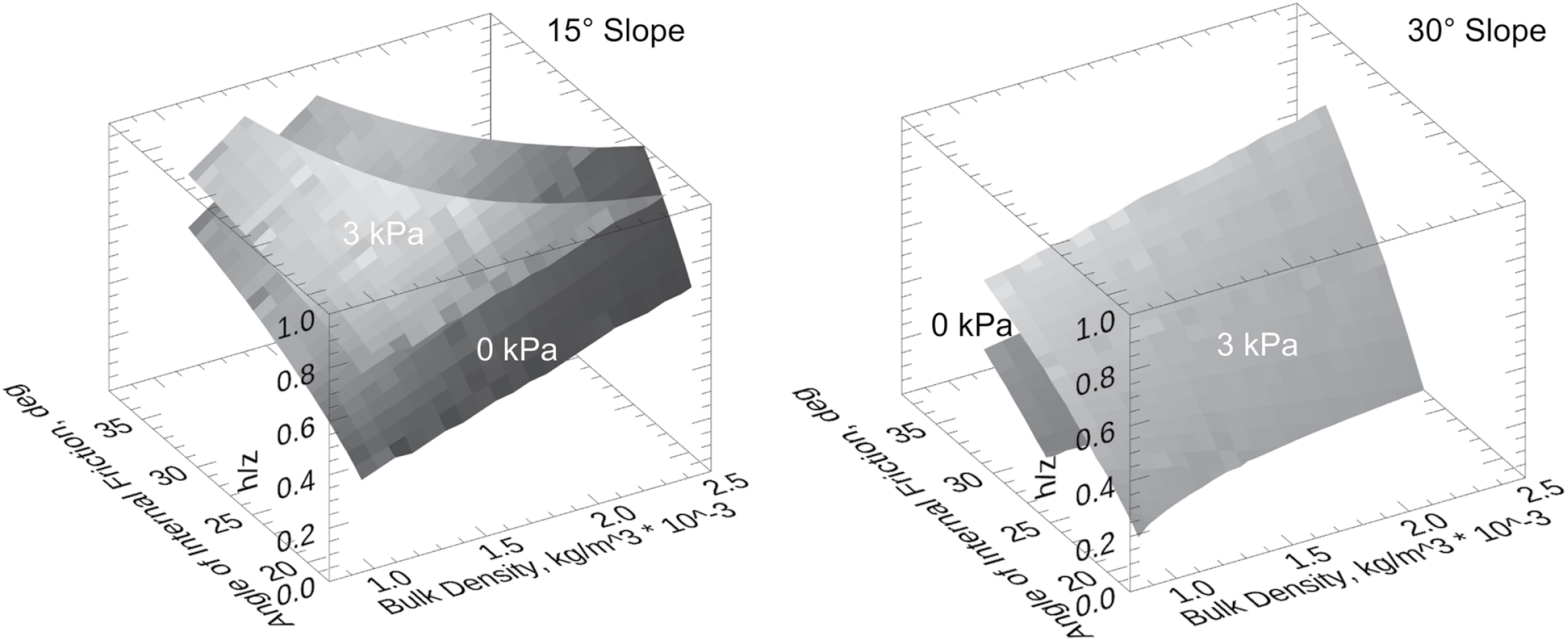
Plot showing the height of the water table, *h*, above the failure plane relative to the thickness of the failed material, *z*, as a function of blocky regolith bulk density and angle of internal friction at the blocky regolith bedrock failure plane. Results were generated using a failure‐plane slope of 15° and 30°. The bottom surface in each plot shows the range for a cohesionless boundary whereas the top surface is for a cohesion of 3 kPa. Note the relatively high h/z values for the low slope case, including values greater than one, which would imply overpressure, exfiltration, or surface water would be required to induce failure. The surfaces are illuminated by a directional light source aligned with the viewer.

For model calculations, we focused on slopes of both 15° and 30°, values in which dry failure would be unlikely for the lower slope and perhaps marginally likely for the higher slope. We used a depth to the failure plane of 6 m, a value consistent with the head scarp relief for SV1. We employed a range of bulk densities, cohesive strengths, and angles of internal friction consistent with the values quoted in the last two paragraphs (Figure 19).

The intent of the modeling was to evaluate the role of pore water in initiating a landslide that would then mobilize into a debris flow, and not to model the likely complex of failure events associated with any of the debris deposits and associated features. Results demonstrate that for the case of no cohesive strength at the failure plane, and for reasonable range of input parameters, the low gradient of 15° requires high values of *h/z* to induce instability. For example, with a bulk density or 1,700 kg/m^3^ and relatively low angle of internal friction of 25°, the *h/z* needed to initiate failure would be 0.86. Note that *h/z* is the fraction of the blocky regolith mass that is saturated. For a cohesive strength of 3 kPa model results for the same bulk density and angle of internal friction, *h/z* would be 1.16, consistent with overpressure that exceeds hydrostatic pressure, exfiltration, and leads to runoff, or perhaps even liquefaction. The likely values of pore water content are well within the terrestrial range that mobilizes landslides into debris flows, and cutting into underlying rocks, thereby generating chutes (Iverson, 2000; Hsu et al., 2011; Stock & Dietrich, 2003). Even at a 30° slope value a significant amount of pore water would be needed to reduce the effective stress and initiate failure for a reasonable range of cohesive strengths and angles of internal friction.

Factors such as localized reduction in down slope saturated conductivity can force shallow groundwater to the surface, with significant exfiltration head gradients (e.g., Wilson & Dietrich, 1987; Wilson et al., 1989). Exfiltration gradients can also occur when groundwater is forced to the surface due to downslope boundary conditions (e.g., Iverson & Reid, 1992; Tóth, 1963). Such processes may be more likely in the case of deep groundwater flow from a more regional scale flow field. Note that Palucis et al. (2016) defined a highest late‐stage lake level that would have put the locations for SV1 and SV2 underwater, with concurrent hydrostatic stresses applied against these locations (e.g., Berilgen, 2007). Lake level drawdown could have removed the confining stresses, and if the drawdown rate exceeded the rate of groundwater flow toward the lake, seepage stresses would have significantly decreased the effective normal stresses, leading to failure. In summary, surface parallel flow, exfiltrating flow driven by conductivity variations or from deeper sources, surface flow in combination with subsurface flow, and seepage pressures generated during lake draw‐down phases, are all viable mechanisms for landslide failures, with enough water to generate erosive debris flows. In contrast, the model without water (*h*/*z* = 0) would require unusually low friction angles to explain failure. And, even if that was the case, it would not explain why failure was localized whereas neighboring canyon walls with similar slope angles do not show evidence for failure. We thus conclude that water was likely a necessary ingredient in initiating wall debris failure and transporting the material downslope, merging with debris on the canyon floors. These processes could have been driven by or enhanced by local precipitation, with water largely sinking into the permeable regolith, overall increasing the rate of systematic downslope movement of material to the floor and down‐canyon.

## 8 Timing and Evolution of the Debris Deposits

The deposition of the sedimentary strata that compose Mount Sharp, and subsequent excavation of the mound‐like shape, were thought to have been largely finished by ∼3.1–3.3 Gya (i.e., during the late Hesperian Era, e.g., Grotzinger et al., 2015). A number of hydrogeomorphic events are thought to have occurred during or shortly thereafter. For example, Sakarya Vallis, Gediz Vallis, and other nearby canyons (Palucis et al., 2016), formed after Mount Sharp was eroded to a similar form as present, or these canyons would not run generally in the downhill direction defined by the shape of mound. The presence of fluvial channels, deltaic complexes, and fans superimposed on the plains surrounding the mound provide evidence for three discrete fluvial‐lacustrine events (Palucis et al., 2014, 2016). As noted by Thomson et al., (2011), Palucis et al., (2016), and Bryk et al., (2019, 2020) the Gediz Vallis ridges were also emplaced during this latter time‐period. Unfortunately, the small areal extent of the debris deposits that are the focus of this paper limits our ability to use impact crater abundances to date them with a more detailed timing. Most likely, the landslide failures, debris flows, and connections to floor debris were coincident with these late‐stage hydrogeomorphic events. In the case of Gediz Vallis the central channel was likely caused by fluvial processes, becoming a ready conduit for down‐canyon transport of debris (Bryk et al., 2020).

We hypothesize that the modification of the debris deposits and chutes through wind erosion has removed much of the evidence of the failure events. Mesoscale atmospheric circulation models show that during the day winds tend to be northwesterly, blowing up the northern side of Mount Sharp, reversing at night, blowing down the northern side of the mound (Newman et al., 2017). Unfortunately, Curiosity's rover environmental monitoring station (REMS) wind directional data are limited temporally and spatially. However, the available data are consistent with these predicted reversing wind directions (Newman et al., 2017; Pla‐Garcia et al., 2016; Rafkin et al., 2016). The yardang orientation within the sulphate‐bearing strata also indicates the dominant winds are northwesterly or southeasterly. The alignment of the slope winds with the northwest‐southeast orientation of Gediz Vallis would channel and enhance these winds. The possibility of downhill mass movement of blocky debris, late‐stage water running down the canyon walls, coupled with channeled and accelerated winds could have preferentially eroded bedrock, removing most of the evidence for landslides and debris flows. We note that the orientation of the linear ridges on the plateaus on either side of Sakarya Vallis are generally consistent with these wind directions and imply significant erosion of bedrock, that is, generation of yardangs (Figure 2). On the other hand, the cross orientation of Sakarya Vallis, as compared to the slope winds, as well as the relief of the canyon (∼350 m compared to ∼75 m at Gediz Vallis), are all consistent with sheltering the canyon floor and walls from the high erosion rates expected for Gediz Vallis.

Over the past ∼3 Gyr, the canyons and debris features have been largely shaped by wind erosion and wall retreat as the regolith migrated downhill. The undulating nature of the floor deposits is interpreted to be a consequence of former locations of wall debris merging with the central floor debris. Possibly, all but the floor debris undulations and small head scarps remain after erosion of the wall deposits.

## 9 Conclusions

Sakarya Vallis on the southwestern side of Mount Sharp exhibits a number of landforms indicative of landslides and debris flows that transferred material from the walls to the floors. Debris on the floors then moved down‐canyon. Infinite slope modeling, coupled with the distinct morphologic features, shows that the best‐preserved wall debris deposits, that is, with head scarps, chutes, and lateral ridges, can be explained as landslide failure due to pore water pressure from groundwater, followed by mobilization as debris flows. The high solubility of the sulphate‐bearing strata led to formation of blocky regolith conditioned to fail in this manner. The presence of numerous debris ridges on the canyon walls implies significant differential wind erosion since the debris accumulated, with the wall strata stripped at a higher rate relative to the more resistant debris deposits. Similar processes operated in Gediz Vallis. In this case, we interpret that the alignment of the canyon with erosive slope winds over several billion years removed all but one wall‐based debris ridge and left a single channel‐filling debris deposit on the canyon floor. Results are consistent with other late‐stage (i.e., late Hesperian Era) hydrogeomorphic events reported previously, including river channels, fans, and deltas in Gale Crater.

## Data Availability

The data sets used in this study are publicly available through the Planetary Data System Geosciences Node (https://pds-geosciences.wustl.edu/). The CTX data used are from Malin (2007). The HiRISE data used are from McEwen (2005, 2006, 2009). The HRSC data used are from the European Space Agency (2013, 2020). The HiRISE mosaic over Gale crater is available at the USGS Annex (Calef & Parker, 2016). The HiRISE‐derived DTMs over Sakarya Vallis are publicly available through the Zenodo data repository (Hughes, 2021). The product IDs and DOIs of the data sets used in this study are also listed in Table 1.

## References

[jgre21816-bib-0001] Anderson, R. B. , & Bell, J. F. (2010). Geologic mapping and characterization of Gale crater and implications for its potential as a Mars Science Laboratory landing site. MARS, 5, 76–128. 10.1555/mars.2010.0004

[jgre21816-bib-0002] Arvidson, R. E. , Anderson, R. C. , Bartlett, P. , Bell, J. F. , Christensen, P. R. , Chu, P. , et al. (2004). Localization and physical properties experiments conducted by opportunity at Meridiani Planum. Science, 306(5702), 1730–1733. 10.1126/science.1104211 15576608

[jgre21816-bib-0003] Arvidson, R. E. , Bell, J. F. , Bellutta, P. , Cabrol, N. A. , Catalano, J. G. , Cohen, J. , et al. (2010). Spirit Mars Rover Mission: Overview and selected results from the northern home plate winter haven to the side of scamander crater. Journal of Geophysical Research, 115(9), 1–19. 10.1029/2010JE003633

[jgre21816-bib-0004] Banham, S. G. , Gupta, S. , Rubin, D. M. , Edgett, K. S. , Barnes, R. , Van Beek, J. , & Vasavada, A. R. (2021). A rock record of complex aeolian bedforms in a hesperian desert landscape: The Stimson formation as exposed in the Murray Buttes, Gale Crater, Mars. Journal of Geophysical Research: Planets, 126(4), e2020JE006554. 10.1029/2020JE006554

[jgre21816-bib-0005] Berilgen, M. M. (2007). Investigation of stability of slopes under drawdown conditions. Computers & Geosciences, 24, 81–91. 10.1016/j.compgeo.2006.10.004

[jgre21816-bib-0006] Bethke, C. M. (2007). Geochemical and biogeochemical reaction modeling (p. 547). Cambridge University Press.

[jgre21816-bib-0007] Bryk, A. B. , Dietrich, W. E. , Fox, V. K. , Bennett, K. A. , Banham, S. G. , Lamb, M. P. , et al. (2020). The stratigraphy of Central and Western butte and the Greenheugh pediment. LPSC abstract 2612.

[jgre21816-bib-0008] Bryk, A. B. , Dietrich, W. E. , Lamb, M. P. , Grotzinger, J. P. , Vasavada, A. R. , Stack, K. M. , et al. (2019). In Curiosity’s path: The geomorphology and stratigraphy of Greenheugh pediment and Gediz Vallis ridge in Gale crater. LPSC abstract 2263.

[jgre21816-bib-0009] Calef, F. J. , & Parker, T. (2016). MSL Gale merged orthophoto mosaic, publisher: PDS Annex, U.S. Geological Survey. Retrieved from https://astrogeology.usgs.gov/search/map/Mars/MarsScienceLaboratory/Mosaics/MSL_Gale_Orthophoto_Mosaic_10m_v3

[jgre21816-bib-0010] Collins, G. S. , & Melosh, H. J. (2003). Acoustic fluidization and the extraordinary mobility of sturzstroms. Journal of Geophysical Research, 108(B10), 1–14. 10.1029/2003jb002465

[jgre21816-bib-0011] Dietrich, W. E. , Bellugi, D. , & Real de Asua, R. (2001). Validation of the shallow landslide model, SHALSTAB, for forest management. Water Science and Application, 2, 195–227. 10.1029/ws002p0195

[jgre21816-bib-0012] European Space Agency . (2013). MEX‐M‐HRSC‐5‐REFDR‐DTM. [Data set]. MEX‐M‐HRSC‐5‐REFDR‐DTM. European Space Agency. 10.5270/esa-1uqwzjv

[jgre21816-bib-0013] European Space Agency . (2020). MEX‐M‐HRSC‐5‐REFDR‐MAPPROJECTED. [Data set]. MEX‐M‐HRSC‐5‐REFDR‐MAPPROJECTED. European Space Agency. 10.5270/esa-pm8ptbq

[jgre21816-bib-0014] Glotch, T. D. , Bandfield, J. L. , Christensen, P. R. , Calvin, W. M. , McLennan, S. M. , Clark, B. C. , & Squyres, S. W. (2006). Mineralogy of the light‐toned outcrop at Meridiani Planum as seen by the miniature thermal emission spectrometer and implications for its formation. Journal of Geophysical Research: Planets, 111(12), 1–14. 10.1029/2005JE002672

[jgre21816-bib-0015] Gorevan, S. P. , Myrick, T. , Davis, K. , Chau, J. J. , Bartlett, P. , Mukherjee, S. , & Richter, L. (2003). Rock abrasion tool: Mars exploration rover mission. Journal of Geophysical Research, 108(E12), 8068. 10.1029/2003je002061

[jgre21816-bib-0016] Grant, J. A. , Wilson, S. A. , Mangold, N. , Calef, F. , & Grotzinger, J. P. (2014). The timing of alluvial activity in Gale crater, Mars. Geophysical Research Letters, 41(4), 1142–1148. 10.1002/2013GL058909

[jgre21816-bib-0017] Grotzinger, J. P. , Arvidson, R. E. , Bell, J. F. , Calvin, W. , Clark, B. C. , Fike, D. A. , & Watters, W. A. (2005). Stratigraphy and sedimentology of a dry to wet eolian depositional system, Burns formation, Meridiani Planum, Mars. Earth and Planetary Science Letters, 240(1), 11–72. 10.1016/j.epsl.2005.09.039

[jgre21816-bib-0018] Grotzinger, J. P. , Gupta, S. , Malin, M. C. , Rubin, D. M. , Schieber, J. , Siebach, K. , & Wilson, S. A. (2015). Deposition, exhumation, and paleoclimate of an ancient lake deposit, Gale crater, Mars. Science, 350(6257), 10.1126/science.aac7575 26450214

[jgre21816-bib-0019] Guimpier, A. , Conway, S. J. , Mangeney, A. , Lucas, A. , Mangold, N. , Peruzzetto, M. , & Cremonese, G. (2021). Dynamics of recent landslides (<20 My) on Mars: Insights from high‐resolution topography on Earth and Mars and numerical modelling. Planetary and Space Science, 206(June), 105303. 10.1016/j.pss.2021.105303

[jgre21816-bib-0020] Gwinner, K. , Scholten, F. , Preusker, F. , Elgner, S. , Roatsch, T. , Spiegel, M. , & Heipke, C. (2010). Topography of Mars from global mapping by HRSC high‐resolution digital terrain models and orthoimages: Characteristics and performance. Earth and Planetary Science Letters, 294(3–4), 506–519. 10.1016/j.epsl.2009.11.007

[jgre21816-bib-0021] Gwinner, K. , Scholten, F. , Spiegel, M. , Schmidt, R. , Giese, B. , Oberst, J. , & Neukum, G. (2009). Derivation and Validation of High‐Resolution digital terrain models from Mars express HRSC data. Photogrammetric Engineering & Remote Sensing, 75(9), 1127–1142. 10.14358/PERS.75.9.1127

[jgre21816-bib-0022] Hoek, E. , & Bray, J. W. (1999). Rock slope engineering (3rd ed.). Institute of Mining and Metallurgy, Taylor and Francis, revised.

[jgre21816-bib-0023] Hsu, L. , Kaitna, R. , Dietrich, W. E. , & Sklar, L. S. (2011). Boundary shear stress of granular flows. In R. Genevois , D. L. Hamilton , & A. Prestininzi (Eds.), Proceedings of the 5th international conference on debris flow hazards mitigation, mechanics, prediction and assessment, Padua, Italy, June 14‐17, 2011. Italian Journal of Engineering Geology and Environment.

[jgre21816-bib-0024] Hughes, M. N. (2021). HiRISE‐generated DTMs over Sakarya Vallis in Gale crater, Mars. [Data set]. Zenodo. 10.5281/ZENODO.5114387

[jgre21816-bib-0025] Hughes, M. N. , Arvidson, R. E. , Bryk, A. B. , Dietrich, W. E. , Lamb, M. P. , & Catalano, J. G. (2020). Mass movements and debris deposits in the Sakarya Vallis and Gediz Vallis, Gale crater, Mars. LPSC abstract 2426.

[jgre21816-bib-0026] Iverson, R. M. (1997). The Physics of Debris flows. Review of Geophysics, 35, 245–296. 10.1029/97rg00426

[jgre21816-bib-0027] Iverson, R. M. (2000). Landslide triggering by rain infiltration. Water Resources Research, 36(7), 1897–1910. 10.1029/2000wr900090

[jgre21816-bib-0028] Iverson, R. M. , & Major, J. J. (1986). Groundwater seepage vectors and the potential for hillslope failure and debris flow mobilization. Water Resources Research, 22(11), 1543–1548. 10.1029/wr022i011p01543

[jgre21816-bib-0029] Iverson, R. M. , & Reid, M. E. (1992). Gravity‐driven groundwater flow and slope failure potential, 1. Elastic effective‐stress model. Water Resources Research, 28(3), 925–938. 10.1029/91wr02694

[jgre21816-bib-0030] Jaumann, R. , Neukum, G. , Behnke, T. , Duxbury, T. C. , Eichentopf, K. , Flohrer, J. , & Wählisch, M. (2007). The high‐resolution stereo camera (HRSC) experiment on Mars Express: Instrument aspects and experiment conduct from interplanetary cruise through the nominal mission. Planetary and Space Science, 55(7–8), 928–952. 10.1016/j.pss.2006.12.003

[jgre21816-bib-0031] Kaitna, R. , Palucis, M. C. , Yohannes, B. , Hill, K. M. , & Dietrich, W. E. (2016). Effects of coarse grain size distribution and fine particle content on pore fluid pressure and shear behavior in experimental debris flows. Journal of Geophysical Research: Earth Surface, 121, 415–441. 10.1002/2015JF003725

[jgre21816-bib-0032] Knuth, M. A. , Johnson, J. B. , Hopkins, M. A. , Sullivan, R. J. , & Moore, J. M. (2012). Discrete element modeling of a Mars Exploration Rover wheel in granular material. Journal of Terramechanics, 49(1), 27–36. 10.1016/j.jterra.2011.09.003

[jgre21816-bib-0033] Le Deit, L. , Hauber, E. , Fueten, F. , Pondrelli, M. , Rossi, A. P. , & Jaumann, R. (2013). Sequence of infilling events in Gale crater, Mars: Results from morphology, stratigraphy, and mineralogy. Journal of Geophysical Research: Planets, 118, 2439–2473. 10.1002/2012JE004322

[jgre21816-bib-0034] Liu, Y. , & Catalano, J. G. (2016). Implications for the aqueous history of southwest Melas Chasma, Mars as revealed by interbedded hydrated sulfate and Fe/Mg‐smectite deposits. Icarus, 271, 283–291. 10.1016/j.icarus.2016.02.015

[jgre21816-bib-0035] Lucchitta, B. K. (1979). Landslides in Valles Marineris, Mars. Journal of Geophysical Research, 84(B14), 8097–8113. 10.1029/jb084ib14p08097

[jgre21816-bib-0036] Lucchitta, B. K. (1987). Valles Marineris, Mars: Wet debris flows and ground ice. Icarus, 72, 411–429. 10.1016/0019-1035(87)90183-7

[jgre21816-bib-0037] Malin, M. C. (2007). MRO Context Camera experiment data record level 0 v1.0. [Data Set]. NASA Planetary Data System. 10.17189/1520266

[jgre21816-bib-0038] Malin, M. C. , Bell, J. F. , Cantor, B. A. , Caplinger, M. A. , Calvin, W. M. , Clancy, R. T. , & Wolff, M. J. (2007). Context camera investigation on board the Mars reconnaissance orbiter. Journal of Geophysical Research, 112, E05S04. 10.1029/2006JE002808

[jgre21816-bib-0039] Mayer, D. P. , & Kite, E. S. (1903). An Integrated Workflow for Producing Digital Terrain Models of Mars from CTX and HiRISE Stereo Data Using the NASA Ames Stereo Pipeline, 47th Lunar and Planetary Science Conference, held March 21‐25, 2016 at The Woodlands, Texas. LPI Contribution No (p. 1241).

[jgre21816-bib-0040] McEwen, A. S. (2005). MRO Mars high resolution image science experiment EDR V1.0. [Data Set]. NASA Planetary Data System. 10.17189/1520179

[jgre21816-bib-0041] McEwen, A. S. (2006). MRO Mars high resolution image science experiment RDR V1.0. [Data Set]. NASA Planetary Data System. 10.17189/1520303

[jgre21816-bib-0042] McEwen, A. S. (2009). MRO Mars high resolution image science experiment DTM V1.0. [Data Set]. NASA Planetary Data System. 10.17189/1520227

[jgre21816-bib-0043] McEwen, A. S. , Eliason, E. M. , Bergstrom, J. W. , Bridges, N. T. , Hansen, C. J. , Delamere, W. A. , & Weitz, C. M. (2007). Mars reconnaissance orbiter’s high resolution imaging science experiment (HiRISE). Journal of Geophysical Research, 112(5), 1–40. 10.1029/2005JE002605

[jgre21816-bib-0044] Milledge, D. G. , Bellugi, D. , McKean, J. A. , Densmore, A. L. , & Dietrich, W. E. (2014). A multidimensional stability model for predicting shallow landslide size and shape across landscapes. Journal of Geophysical Research: Earth Surface, 119, 2481–2504. 10.1002/2014jf003135 26213663 PMC4508911

[jgre21816-bib-0045] Milliken, R. E. , Grotzinger, J. P. , & Thomson, B. J. (2010). Paleoclimate of Mars as captured by the stratigraphic record in Gale crater. Geophysical Research Letters, 37, L04201. 10.1029/2009GL041870

[jgre21816-bib-0046] Moore, H. J. , Hutton, R. E. , Clow, G. D. , & Spitzer, C. R. (1987). Physical Properties of the Surface Materials at the Viking Landing Sites on Mars, 222pp. *U. S.Geol. Survey Prof. Pap. 1389*. U. S. Government Printing Office. 10.3133/pp1389

[jgre21816-bib-0047] Morgan, P. , Grott, M. , Knapmeyer‐Endrun, B. , Golombek, M. , Delage, P. , Lognonné, P. , & Kedar, S. (2018). A pre‐landing assessment of regolith properties at the InSight landing site. Space Science Reviews (Vol. 214). Springer Nature B.V. 10.1007/s11214-018-0537-y

[jgre21816-bib-0048] Nahm, A. L. , & Schultz, R. A. (2007). Outcrop‐scale physical properties of Burns formation at Meridiani Planum, Mars. Geophysical Research Letters, 34, L20203. 10.1029/2007GL031005

[jgre21816-bib-0049] Newman, C. E. , Gómez‐Elvira, J. , Marin, M. , Navarro, S. , Torres, J. , Richardson, M. I. , et al. (2017). Winds measured by the rover environmental monitoring station (REMS) during the Mars science laboratory (MSL) rover’s bagnold dunes campaign and comparison with numerical modeling using MarsWRF. Icarus, 291, 203–231. 10.1016/j.icarus.2016.12.016 30393391 PMC6208171

[jgre21816-bib-0050] Okura, Y. , Kitahara, H. , Sammori, T. , & Kawanami, A. (2000). Effects of rockfall volume on runout distance. Engineering Geology, 58(2), 109–124. 10.1016/S0013-7952(00)00049-1

[jgre21816-bib-0051] Pain, C. F. , Clarke, J. D. A. , & Thomas, M. (2007). Inversion of relief on Mars. Icarus, 190(2), 478–491. 10.1016/j.icarus.2007.03.017

[jgre21816-bib-0052] Palucis, M. C. , Dietrich, W. E. , Hayes, A. G. , Williams, R. M. E. , Gupta, S. , Mangold, N. , & Sumner, D. Y. (2014). The origin and evolution of the Peace Vallis fan system that drains to the Curiosity landing area, Gale crater, Mars. Journal of Geophysical Research: Planets, 119, 705–728. 10.1002/2013JE004583

[jgre21816-bib-0053] Palucis, M. C. , Dietrich, W. E. , Williams, R. M. E. , Hayes, A. G. , Parker, T. , Sumner, D. Y. , & Newsom, H. (2016). Sequence and relative timing of large lakes in Gale crater (Mars) after the formation of Mount Sharp. Journal of Geophysical Research: Planets, 121, 472–496. 10.1002/2015JE004905.Received

[jgre21816-bib-0054] Pla‐Garcia, J. , Rafkin, S. C. R. , Kahre, M. , Gomez‐Elvira, J. , Hamilton, V. E. , Navarro, S. , et al. (2016). The meteorology of Gale crater as determined from rover environmental monitoring station observations and numerical modeling. Part I: Comparison of model simulations with observations. Icarus, 280, 103–113. 10.1016/j.icarus.2016.03.013

[jgre21816-bib-0055] Prancevic, J. P. , Lamb, M. P. , & Fuller, B. M. (2014). Incipient sediment motion across the river to debris‐flow transition. Geology, 42(3), 191–194. 10.1130/G34927.1

[jgre21816-bib-0056] Prancevic, J. P. , Lamb, M. P. , Palucis, M. C. , & Venditti, J. G. (2018). The role of three‐dimensional boundary stresses in limiting the occurrence and size of experimental landslides. Journal of Geophysical Research: Earth Surface, 123, 56–65. 10.1002/2017JF004410

[jgre21816-bib-0057] Quantin, C. , Allemand, P. , Mangold, N. , & Delacourt, C. (2004). Ages of Valles Marineris (Mars) landslides and implications for canyon history. Icarus, 172(2), 555–572. 10.1016/j.icarus.2004.06.013

[jgre21816-bib-0058] Rafkin, S. C. R. , Pla‐Garcia, J. , Kahre, M. , Gomez‐Elvira, J. , Hamilton, V. E. , Marín, M. , et al. (2016). The meteorology of Gale crater as determined from rover environmental Monitoring station observations and numerical modeling. Part II: Interpretation. Icarus, 280, 114–138. 10.1016/j.icarus.2016.01.031

[jgre21816-bib-0059] Selby, M. J. (1993). Water in soils and hillslope hydrology. Hillslope Materials and Processes. Oxford University Press.

[jgre21816-bib-0060] Shaw, A. , Arvidson, R. E. , Bonitz, R. , Carsten, J. , Keller, H. U. , Lemmon, M. T. , & Trebi‐Ollennu, A. (2009). Phoenix soil physical properties investigation. Journal of Geophysical Research, 114(12), 1–19. 10.1029/2009JE003455

[jgre21816-bib-0061] Sheppard, R. Y. , Milliken, R. E. , Parente, M. , & Itoh, Y. (2020). Updated perspectives and hypotheses on the mineralogy of lower Mt. Sharp, Mars, as seen from orbit. Journal of Geophysical Research: Planets, 126(2), e2020JE006372. 10.1029/2020je006372

[jgre21816-bib-0062] Stock, J. , & Dietrich, W. E. (2003). Valley incision by debris flows: Evidence of a topographic signature. Water Resources Research, 39(4), 1089. 10.1029/2001WR001057

[jgre21816-bib-0063] Sullivan, R. , Anderson, R. , Biesiadecki, J. , Bond, T. , & Stewart, H. (2011). Cohesions, friction angles, and other physical properties of Martian regolith from Mars Exploration Rover wheel trenches and wheel scuffs. Journal of Geophysical Research, 116(2), E02006. 10.1029/2010JE003625

[jgre21816-bib-0064] Thomson, B. J. , Bridges, N. T. , Cohen, J. , Hurowitz, J. A. , Lennon, A. , Paulsen, G. , & Zacny, K. (2013). Estimating rock compressive strength from Rock Abrasion Tool (RAT) grinds. Journal of Geophysical Research: Planets, 118, 1233–1244. 10.1002/jgre.20061

[jgre21816-bib-0065] Thomson, B. J. , Bridges, N. T. , Milliken, R. , Baldridge, A. , Hook, S. J. , Crowley, J. K. , & Weitz, C. M. (2011). Constraints on the origin and evolution of the layered mound in Gale crater, Mars using Mars reconnaissance orbiter data. Icarus, 214(2), 413–432. 10.1016/j.icarus.2011.05.002

[jgre21816-bib-0066] Tóth, J. (1963). A theoretical analysis of groundwater flow in small drainage basins. Journal of Geophysical Research, 68(16), 4795–4812.

[jgre21816-bib-0067] Wald, J. A. , Graham, R. C. , & Schoeneberger, P. T. (2012). Distribution and properties of soft weathered bedrock at ≤ 1m depth in the contiguous United States (p. 1131). Publications from USDA‐ARS/UNL Faculty.

[jgre21816-bib-0068] Williams, R. M. E. , Chidsey, T. C. , & Eby, D. E. (2007). Exhumed paleochannels in central Utah–Analogs for raised curvilinear features on Mars. In G. C. Willis , M. D. Hylland , D. L. Clark , & T. C. Chidsey Jr . (Eds.), Central Utah–diverse geology of a dynamic landscape (Vol. 36, pp. 220–235). Utah Geological Association Publication.

[jgre21816-bib-0069] Wilson, C. J. , & Dietrich, W. E. (1987). The contribution of bedrock groundwater flow to storm runoff and high pore pressure development in hollows, Proc. Int. Symp. on Erosion and Sedimentation in the Pacific Rim, 3‐7 August 1987, Corvallis, Ore (Vol. 165, pp. 49–59). Int. Assoc. Hydrological Sciences Bull., Pub.

[jgre21816-bib-0070] Wilson, C. J. , Dietrich, W. E. , & Narasimhan, T. N. (1989). Predicting high pore pressures and saturation overland flow in unchannelled hillslope valleys, Hydrology and Water Resources Symposium (pp. 392–396). Institution of Engineering Australia.

[jgre21816-bib-0071] Zaki, A. S. , Pain, C. F. , Edgett, K. S. , & Castelltort, S. (2021). Global inventory of fluvial ridges on Earth and lessons applicable to Mars. Earth‐Science Reviews, 216. 10.1016/j.earscirev.2021.103561

